# Protocol for the Wessex AsThma CoHort of difficult asthma (WATCH): a pragmatic real-life longitudinal study of difficult asthma in the clinic

**DOI:** 10.1186/s12890-019-0862-2

**Published:** 2019-05-24

**Authors:** Adnan Azim, Heena Mistry, Anna Freeman, Clair Barber, Colin Newell, Kerry Gove, Yvette Thirlwall, Matt Harvey, Kimberley Bentley, Deborah Knight, Karen Long, Frances Mitchell, Yueqing Cheng, Judit Varkonyi-Sepp, Wolfgang Grabau, Paddy Dennison, Hans Michael Haitchi, S. Hasan Arshad, Ratko Djukanovic, Tom Wilkinson, Peter Howarth, Ramesh J. Kurukulaaratchy

**Affiliations:** 1 Clinical and Experimental Sciences, Faculty of Medicine, University of Southampton, Southampton, UK; 2 National Institute for Health Research (NIHR) Southampton Biomedical Research Centre at University Hospital Southampton NHS Foundation Trust, Southampton, UK; 3 Institute for Life Sciences, University of Southampton, Southampton, UK; 4 Asthma Allergy and Clinical Immunology Department, University Hospital Southampton NHS Foundation Trust, Southampton, UK; 5 The David Hide Asthma & Allergy Research Centre, St Mary’s Hospital, Newport, Isle of Wight UK; 6 Patient Involvement Group, Southampton, UK; 7 Respiratory Medicine & Allergy, Asthma, Allergy & Clinical Immunology, Mailpoint 52, Floor 2 Minerva House, Southampton General Hospital, Tremona Road, Southampton, Hampshire SO16 6YD UK

**Keywords:** Asthma, Difficult Asthma, Severe Asthma, Prospective, Longitudinal, Cohort, Biobank

## Abstract

**Background:**

Asthma is now widely recognised to be a heterogeneous disease. The last two decades have seen the identification of a number of biological targets and development of various novel therapies. Despite this, asthma still represents a significant health and economic burden worldwide. Why some individuals should continue to suffer remains unclear.

**Methods:**

The Wessex Asthma Cohort of Difficult Asthma (WATCH) is an ongoing ‘real-life’, prospective study of patients in the University Hospital Southampton Foundation Trust (UHSFT) Difficult Asthma service. Research data capture is aligned with the extensive clinical characterisation required of a commissioned National Health Service (NHS) Specialist Centre for Severe Asthma. Data acquisition includes detailed clinical, health and disease-related questionnaires, anthropometry, allergy and lung function testing, radiological imaging (in a small subset) and collection of biological samples (blood, urine and sputum). Prospective data are captured in parallel to clinical follow up appointments, with data entered into a bespoke database.

**Discussion:**

The pragmatic ongoing nature of the WATCH study allows comprehensive assessment of the real world clinical spectrum seen in a Specialist Asthma Centre and allows a longitudinal perspective of deeply phenotyped patients. It is anticipated that the WATCH cohort would act as a vehicle for potential collaborative asthma studies and will build upon our understanding of mechanisms underlying difficult asthma.

**Electronic supplementary material:**

The online version of this article (10.1186/s12890-019-0862-2) contains supplementary material, which is available to authorized users.

## Background

Over 300 million people are affected by asthma worldwide, including an estimated 1 in 12 adults in the United Kingdom (UK), half of whom will experience severe symptoms at some point in their lives [1]. “Severe asthma” is defined by the ERS/ATS guidelines as asthma requiring treatment with high-dose inhaled corticosteroid/long-acting β-agonist, plus a second controller and/or oral corticosteroids to prevent it from becoming uncontrolled; or asthma that remains uncontrolled despite this therapy [2]. In contrast, “difficult to control” or “difficult to manage asthma”, also commonly known as “difficult asthma”, is defined as problematic asthma despite treatment with high-dose inhaled therapies and/or frequent or continuous use of oral corticosteroids, where poor asthma control may be related to other factors such as poor medication adherence, significant co-morbidities (including rhinitis, gastro-oesophageal reflux, obesity, structural lung disease and psychological co-morbidities) and external triggers (e.g. allergens and environmental factors) [3]. Difficult asthma is characterised by regular exacerbations, poor quality of life and increased morbidity/mortality, which is thought to account for the majority of asthma related health expenditure [4–6]. Its cost to the National Health Service (NHS) is estimated at £1 billion/year in addition to the hidden societal costs of disability, missed schooling and lost work days [7]. This is more expensive per patient than some other chronic diseases, such as Stroke and COPD [8].

The management of patients with difficult asthma typically requires escalation of treatment towards a heavy reliance on corticosteroids [9, 10]. Not only is this associated with further morbidity [11], but it is increasingly recognised that not all patients respond to corticosteroids [12]. Clinical diversity is seen across paediatric [13, 14], adolescent [15] and adult populations [16, 17]. Additionally, the stratified prescription of newer biological monoclonal antibody therapies [18, 19] point towards an underlying pathophysiological heterogeneity [20]. Understanding these mechanisms and identifying associated biomarkers would facilitate drug discovery and the stratification of patients towards targeted interventions; “precision medicine” [21, 22].

To this end, the National Institutes of Health (NIH) and National Heart, Lung and Blood Institute (NHLBI) have supported the characterisation of severe asthma with the Severe Asthma Research Program (SARP) cohorts [23], whilst the British Thoracic Society (BTS) Difficult Asthma Network has provided a unique UK perspective drawn from clinical practice [8, 24, 25]. The Unbiased Biomarkers in Prediction of Respiratory Disease Outcomes (U-BIOPRED) pan-European collaboration between academia, industry, patient groups and charities have also aimed to characterise severe asthma, with a specific aim to apply a systems biology approach to generate phenotype “handprints” [26].

Despite our improving understanding of asthma and growing access to novel advanced treatment options (biological therapies and bronchial thermoplasty), mortality from asthma in the UK has remained static since 2001 [27, 28]. Consequently, there has been significant restructuring of NHS Asthma Services to address this unmet clinical need as well as manage high cost novel therapies. In 2013, a selection of specialist asthma services in England were designated as Regional Centres by NHS England (NHSE) [7]. The aim was for these centres to ensure that all complex adult asthma patients receive the same high quality standard of care, that expensive therapies are issued responsibly and an accurate disease register is maintained to link to the BTS Difficult Asthma Registry [8]. In this context, University Hospital Southampton Foundation Trust (UHSFT) has a historical role as a tertiary centre for severe asthma care in southern England and allied to the University of Southampton, which has a longstanding pedigree in world class asthma research, it was designated a Commissioned Specialist Clinical Centre for Severe Asthma, Allergy & Clinical Immunology in 2017.

The Wessex Asthma Cohort of difficult asthma (WATCH) is an ongoing prospective cohort study supported by the National Institute for Health Research (NIHR) Southampton Biomedical Research Centre (BRC), which takes advantage of the extensive clinical characterisation required of an NHSE Specialist Centre to build upon the characterisation efforts described by SARP, U-BIOPRED and the BTS Difficult Asthma Network in the specific context of difficult asthma. Patients, through their clinical care, are given the opportunity to enrol onto the study through a process harmonising research participation and clinical care in alignment with the NHSE 5 year Research and Development Strategy that “Research is Everybody’s Business” [29]. By capturing the longitudinal clinical data available through a Specialist Clinic, WATCH provides a pragmatic opportunity to create a sustainable longitudinal “real-life” cohort of patients with difficult asthma through an operational model that combines standard clinical care and acquisition of research data within single clinic visits.

### Primary objectives

- Development of a real-time “parent” database of well-characterised difficult asthma patients through ongoing enrolment and long-term follow-up aligned to clinical care.
- Cross-sectional phenotypic characterisation of a cohort of difficult asthma patients to describe the disease heterogeneity seen in clinical practice (primary endpoint).
- Longitudinal follow up of this cohort to describe the natural history, intervention responsiveness and long-term prognosis of difficult asthma (secondary endpoints).

### Secondary objectives

- Biobanking of samples to facilitate an understanding of the pathophysiological mechanisms associated with these well-characterised patients (endotypes).
- Invitation of well-characterised patients to novel collaborative (“offspring”) observational studies to further assess their characterised phenotype/endotypes.
- Invitation of well-characterised patients to participate in future novel collaborative (“offspring”) interventional studies to assess response to therapy.

## Methods

### UHSFT Asthma clinical service (Fig. 1)

The UHSFT Adult Asthma multidisciplinary team (MDT) is comprised of Consultants, Research Fellows, Specialist Nurses, Associate Practitioners, Clinical Psychologists, Physiotherapists and Dietitians. The service is provided via a dedicated Regional Severe/Difficult Asthma Clinic, Transitional/Young Asthma Patient Clinic, Isle of Wight Outreach Asthma Clinic, Biologics (Omalizumab, Mepolizumab, Reslizumab and Benralizumab) Clinic, Nurse-led Asthma Clinic, Clinical Psychology Clinic and Respiratory Physiotherapy Clinic. The team has a close working relationship with Allergy and Immunology services, Ear Nose & Throat (ENT) Department (providing ready access to Speech Therapy services), and Gastroenterology (GI) Department (providing ready access to Upper GI physiology testing plus dedicated medical/surgical MDT meetings). Support is also provided by UHSFT Pathology, Radiology and Respiratory Physiology Departments. Patients attending the UHSFT Adult or Transitional Regional Asthma Clinics are assessed by a physician and referred onto further MDT members and investigations, with clinical decisions supported by a weekly post-clinic MDT and monthly Biologics Referral MDT. This facilitates the extensive characterisation of each patient, which in turn enables the clinic to meet the standards of care described by NHSE.

**Fig. 1:**
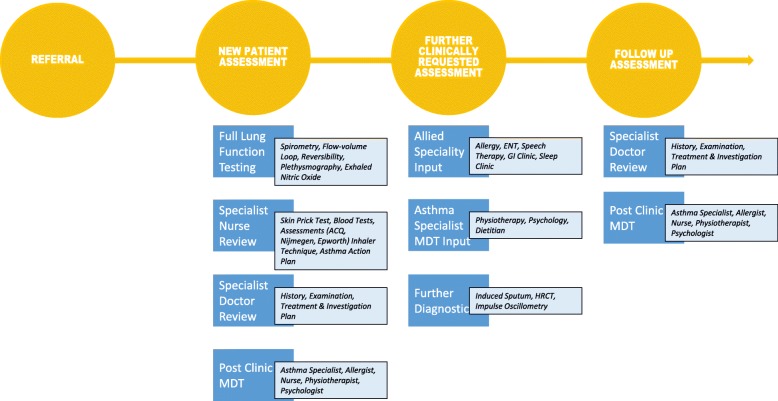
UHSFT Difficult Asthma Service Pathway. A schematic outline of the standard clinical assessment and management pathway in the UHSFT (University Hospital Southampton Foundation Trust) Difficult Asthma Clinic

### WATCH study design (Fig. 2)

**Fig. 2:**
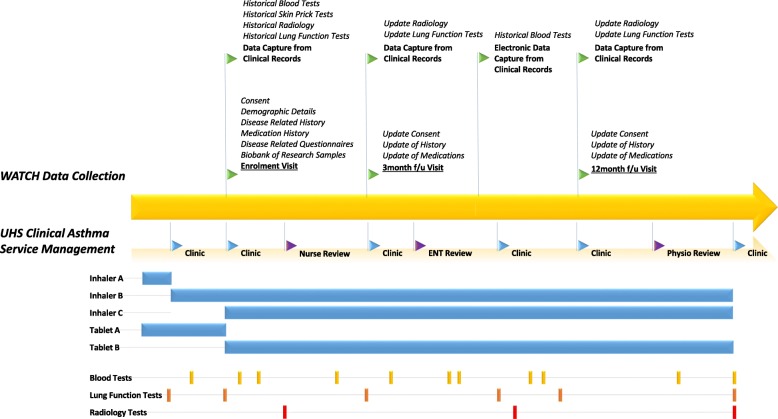
Patient journey with parallel clinical and research activity. A schematic outline of the aligned clinical and research pathways/timelines followed by a patient under the UHSFT Difficult Asthma Clinic who is participating in the WATCH study. The illustration demonstrates examples of how clinical tests (blood tests, lung function and radiology) and medication changes over time may be captured for the study

WATCH is a prospective observational study of patients with difficult asthma attending the Difficult Asthma Clinic at UHSFT. The study is planned as a long-term research vehicle with no limit to enrolment or any time defined end. The study design, protocol and paperwork have been approved by West Midlands – Solihull Research Ethics Committee (REC reference: 14/WM/1226). Patients are recruited into the study by way of a discrete “Enrolment” Study Visit capturing core demographic and clinical information and the results of the characterisation process provided by the clinic. For new to clinic patients, an additional 3 month follow up visit is also undertaken. Critically, the standard clinical assessment for the immediate needs of management and the additional assessments for research needs are collected in sequence, with combined support from Trust staff and research personnel of the NIHR BRC. Thereafter, their records are continuously updated through annual “Follow Up” Study Visits and extraction from their electronic clinical records using the combined hospital clinical record system and research data collection resources in the BRC. This pragmatic, opportunistic approach to data collection takes full advantage of the broad multidisciplinary clinical approach to difficult asthma management without becoming too onerous for the patient, clinician or researcher (Fig. 2). Patients can complete all of their case report forms (CRF) during their clinic appointments for their convenience, but are also given the opportunity to complete their longer enrolment visit on a separate day if more convenient.

### Setting

This is a dual centre study (UHSFT and a satellite outreach clinic on the Isle of Wight) that started recruitment in August 2015. The Difficult Asthma Clinic at UHSFT is a formally commissioned Regional Centre for Severe Asthma, providing support for patients across the South Central England region. At the end of 2017, the clinic looked after 922 patients, of which 459 (49.8%) were “tertiary patients” referred from other local hospitals.

### Recruitment

All patients attending the UHSFT Difficult Asthma Clinic (newly referred and under existing follow up) are approached for enrolment into the WATCH study. In the past 5 years, the clinic has seen an average of 182 new referrals per year, with tertiary referrals accounting for just under 50% of the workload. Based on new referral rates to the clinic, plus the high proportion of the clinic population who met inclusion criteria, an expected minimum study recruitment rate of 100 new patients per year was anticipated for the first 5 years of the study. At the end of 2017, 375 patients had been enrolled. Study enrolment will be continual into the future aligned to the ongoing function of the clinical service with no discrete recruitment target or endpoint.

### Inclusion criteria

- All patients who attend the Adult or Transitional Regional Asthma Clinic at UHSFT or satellite outreach clinics on the Isle of Wight and are managed with “high dose therapies” and/or “continuous or frequent use of oral steroids”, according to the BTS Adult Asthma Management Guidelines 2016.
- Be able to provide informed consent.

### Exclusion criteria

- Patients who attend the Adult or Transitional Regional Asthma Clinic at UHSFT or satellite outreach clinics on the Isle of Wight but are *not* managed with “high dose therapies” and/or “continuous or frequent use of oral steroids”, according to the BTS Adult Asthma Management Guidelines 2016.

### Data collection

Data for the WATCH study are captured both through CRFs completed opportunistically during outpatient appointments and/or discrete study visits, as well as, extraction from hospital electronic clinical record systems after enrolment.

### Enrolment data collection

The initial enrolment CRF contains a large suite of questions that mirrors the extensive characterisation undertaken in clinical practice (summarised in Tables 1 and 2; detailed in Additional file 1). This is completed after the patient has received and read a patient study information sheet, a clinical or research member of the WATCH study team has received consent from the participant, and they have been assigned a study number. A clinical or research member of the WATCH team will then proceed to interview the participant, asking the questions from the Enrolment CRF (detailed in Additional file 1) and then filling in various health and disease related questionnaires with the participant (Table 2).

**Table 1:** Enrolment CRF Data Collection

Data Collection Headings	Including
Demographics	Sex, date of birth, ethnicity, working status, occupation
Disease Related History	Age of asthma onset, family history, atopic history, smoking status, asthma related healthcare utilisation
Environmental Exposures	Pets, moulds, pollens, drugs, other triggers
Details of Co-morbidities	Rhinitis, eczema, chronic obstructive pulmonary disease, bronchiectasis, gastro-oesophageal reflux disease, dysfunctional breathing, vocal cord dysfunction, psychiatric comorbidity
Treatment	Medication lists, Medication Adherence Report Scale for Asthma (MARS-A), allied health professional referrals

**Table 2:** Enrolment CRF Disease Related Questionnaires

Disease Related Questionnaires	Including
Asthma	Asthma Control Questionnaire (ACQ6 & 7), St George’s Respiratory Questionnaire (SGRQ), 5-level version of EuroQoL-5QD (EQ-5D-5 L)
Sleep	Epworth Sleepiness Score
Dysfunctional Breathing	Nijmegen Score
Psychological	Hospital Anxiety and Depression Score (HADS), Mindful Attention Awareness Scale (MAAS), Attention Control Scale (ACS)
GORD	Hull Cough Hypersensitivity Questionnaire
Rhinitis	Sino-Nasal Outcome Test-22 (SNOT-22)
Nutrition	Council on Nutritional Appetite Questionnaire Score (CNAQ), Malnutrition Universal Screening Test (MUST) score

Should the participant not have time to complete all questionnaires at the enrolment visit, then they are completed at the subsequent 3 month follow up (if applicable) or annual follow up visits. They are dated with the date they were completed.

In addition to the questionnaires, objective measures and biological samples (Table 3) are collected during the enrolment process, with the latter being processed by the BRC scientists and technical staff.

**Table 3:** Enrolment CRF Objective Measures

Investigations	Including
Blood Tests	Full blood count, serum total IgE, (as well as any other clinically requested samples)
Lung Function Test	Spirometry +/− reversibility (as well as any other clinically requested tests), fractional exhaled nitric oxide (FeNO)
Anthropometry	Height, weight, body mass index (BMI), bioelectrical impedance
Biobank Samples	Blood, urine, induced sputum, bronchoalveolar lavage (BAL), bronchial brushings and biopsies

Finally, additional objective clinical data from the hospital electronic systems are harnessed to provide retrospective and current investigation findings (Table 4).

**Table 4:** Historical Clinical Record Data Collection

Investigations	May Include	Limit of Data Retrieval
Historical Blood Tests	Full blood count (FBC), Total immunoglobulins E, G, M, A (IgE, IgG, IgM, IgA), Aspergillus precipitins (IgG), 25-hydroxy-vitamin D3, Anti-neutrophil cytoplasmic antibody (ANCA), Antinuclear antibody (ANA), Alpha-1-antitrypsin level (A1AT), Urea & electrolytes, Liver profile, Parathyroid hormone (PTH), Thyroid function tests (Thyroid stimulating hormone & Free thyroxine)	10 years
Allergy Testing	Either Skin prick tests or Specific IgE blood tests to common aeroallergens [Aspergillus fumigatus; Alternaria tenius; Cladosporium; Penicillium; Mixed moulds; Grass mix, Birch & Weed mix pollens; Dermatophagoides pteronyssinus and Dermatophagoides farinae; Feathers; Cat & Dog fur; Horse & Rabbit]	10 years
Radiology	Computed Tomography (CT) or High Resolution CT Chest (HRCT), CT sinuses, Dual Energy X-ray Absorptiometry (DEXA) scan	10 years
Oesophageal Investigation Results & History of Interventions	Oesophagogastroduodenoscopy (OGD), Oesophageal Manometry, pH/Impedance testing & surgical history	10 years
ENT Investigation Results & History of Interventions	Nasoendoscopy & surgical history	10 years
Lung Function Tests	Spirometry +/− bronchodilator reversibility	1 year
	Fractional exhaled nitric oxide (FeNO)	1 year
	Gas transfer	5 years
	Impulse oscillometry	1 year
	Static lung volumes	1 year
	Multiple nitrogen breath washout	1 year

### Follow up data collection

Where patients are enrolled into the study on their initial visit to the Difficult Asthma clinic, they are likely to have a number of clinically requested investigations and changes in therapy. These patients are therefore invited to complete a 3 month post-enrolment CRF to capture any clinical changes during this period. All patients (regardless of whether they are new to clinic or not) are invited to complete Annual CRFs (Additional file 2). Both the 3 month and annual CRFs are more concise than the enrolment CRF. The annual CRF is repeated every year throughout the period of clinical observation and study participation. These CRFs aim to capture an update of asthma and co-morbid disease related information and objective markers (Table 5). Questionnaires completed at enrolment and found to be scored outside ‘normal’ range are repeated at these assessments.

**Table 5:** Longitudinal WATCH data collection

Description	Method
Repeat/Update of Asthma & Co-morbid Disease Related History	Face to face
Repeat/Update of Asthma & Co-morbid Disease Relevant Investigations Performed for Clinical Purposes	Face to face and Electronic capture
Repeat/Update of Treatment	Face to face
Repeat Disease Related Questionnaires (ACQ and EuroQoL-5QD)	Face to face
Repeat Lung Function Test (FeNO, Spirometry +/− other clinically requested tests)	Face to face and Electronic capture
Blood Test Results (FBC, Total IgE +/− other clinically requested tests)	Face to face and Electronic capture
Anthropometry (height, weight, body composition)	Face to Face
Radiological Investigation Results	Electronic capture

Electronic clinical records are reviewed for any new or repeat investigations since the last CRF. These results are then extracted into the study database. If there are any outstanding clinically requested blood tests or lung function tests then these can be performed as part of the study visit.

### Clinical data collection

The WATCH database captures results from clinically requested and processed investigations. These are performed by hospital departments in line with Standard Operating Procedures (SOPs) that conform to standards required of an NHS Hospital Service. A more detailed description of these investigations and departmental standards are provided in Additional file 3: Appendix A

Height and weight are measured by the study team from which BMI is calculated. Additionally, a Medical Body Composition Analyser (SECA, Hamburg Germany) is used to analyse body composition of adults based on Bioelectrical Impedance Analysis (BIA).

Clinically requested lung function tests are performed by the UHSFT Respiratory Physiology Department or Specialist Asthma Nurses, who operate in accordance with local department SOPs and the Association of Respiratory Technology and Physiology (ARTP) guidelines. Some lung function tests are repeated by the BRC research staff at study visits using local SOPs in accordance with ERS/ATS guidelines [30]. A more detailed description of lung function methodology is provided in Additional file 3: Appendix B.

### Biobanking of samples

At enrolment blood and urine is stored for biobanking. If a patient requires sputum induction or bronchoscopy for clinical purposes, they are asked to consent to additional samples being taken for the WATCH biobank, supported by the BRC. A more detailed description of biobanking methodology is provided in Additional file 3: Appendix C.

### Data management

The data model used for WATCH tries, where possible, to adopt a truly longitudinal approach. Change over time is treated explicitly. In this model patients can be enrolled at any time during their clinical journey, and equally may also exit (via clinical discharge, death, or being lost to follow up) at any time. Between those two dates (enrolment and exit or last visit), they are regarded as being ‘under observation’. Data recorded on the CRFs are entered into a bespoke WATCH study database housed by the Southampton BRC. Study status reports, follow up CRFs that highlight areas of missing information/data and discrete datasets can be readily generated. The data capture is categorised as an episode or snapshot. Each CRF, questionnaire or investigation is considered a snapshot. Medication, pregnancy or inpatient stays are considered episodes within the observed period. Where the start date of existing medication is ambiguous or continues past the period of observation, episodes are considered left censored or open-ended respectively. All investigations added to the database are marked by the date of investigation.

Analysis of the whole dataset will be both cross-sectional and longitudinal according to specific study questions. Specific datasets can be extracted from the database to investigate specific hypotheses.

This cohort can also be interrogated for inclusion criteria towards additional trials occurring in parallel to the WATCH study. Participation in clinical trials will not exclude patients from the cohort, but may omit patients from some analysis depending upon the research question.

### Statistical analysis

The primary objective of the “parent” WATCH study is a cross-sectional characterisation of a cohort of difficult asthma patients with secondary objectives including further endotypic and longitudinal characterisation. Data from around the participants’ enrolment and of later follow up can be extracted from the database into discrete datasets to satisfy these aims. Statistical analysis will be performed primarily in IBM SPSS 25 (NY, USA). Categorical data will be presented as counts and percentages, with differences between groups tested by Pearson χ^2^. Continuous data with Gaussian distributions will be presented as mean and standard deviation, whilst continuous data with skewed distributions will be presented as median and interquartile ranges. Differences between groups will be measured by *t*-test and Wilcoxon rank-sum test for Gaussian and skewed continuous data respectively. Where more unbiased assessment of phenotypes is undertaken, techniques such as cluster analysis and topological data analysis will be deployed.

## Results

The quantity and range of data acquired through the WATCH model of data collection is illustrated for the first 410 patients enrolled (Table 6).

**Table 6:** Data Acquisition for the WATCH 410 Cohort

Summary Enrolment Data	Data Acquisition
Demographics	410 (100.0%)
Anthropometry	399 (97.3%)
Asthma History	409 (99.8%)
Asthma Therapy	407 (99.2%)
Smoking History	409 (99.8%)
Co-morbid Disease History	410 (100.0%)
Questionnaires	
*ACQ-6*	371 (90.5%)
*Hospital Anxiety & Depression Score (HADS)*	330 (80.5%)
*Nijmegen*	289 (70.5%)
*Sino-Nasal Outcome Score-22 (SNOT-22)*	250 (61.0%)
*Hull Cough Hypersensitivity*	288 (70.2%)
*EuroQoL-5QD*	72 (17.6%)
*Epworth Sleepiness Score (ESS)*	332 (81.0%)
*Medication Adherence Reporting Scale for Asthma (MARS-A)*	278 (67.8%)
*Attention Control Score (ACS)*	357 (87.1%)
*Mindful Attention Awareness Scale (MAAS)*	270 (65.9%)
Blood Tests	
*Peripheral eosinophil count*	401 (97.8%)
*Total IgE*	399 (97.3%)
*Aspergillus-specific IgE*	378 (92.2%)
*Aspergillus precipitins IgG*	379 (92.4%)
Lung Function Tests	
*Pre-bronchodilator spirometry*	242 (59.0%)
*Post-bronchodilator spirometry*	308 (75.1%)
*Gas transfer*	234 (57.0%)
*Plethysmography*	271 (66.1%)
*Fractional exhaled nitric oxide (FeNO)*	307 (74.9%)
Radiological Imaging	
*High-resolution CT Chest*	262 (63.9%)
*CT Sinuses*	53 (12.9%)
*DEXA Scan*	127 (31.0%)
Biobank Samples	
*Bloods*	331 (80.7%)
*Urine*	298 (72.7%)

## Discussion

In difficult asthma, disease heterogeneity and high prevalence of numerous co-morbidities demands a structured characterisation process to guide optimal clinical care. In the UK, this practice is reinforced by a public NHS, which has evolved a system of specialist centre care for complex conditions, such as difficult asthma, to responsibly gate keep high cost therapies and maintain specialist follow up care. Such a system naturally lends itself to a research facing element. The WATCH study has been established to align clinical practice with the formation of a prospective research cohort reflective of real-life clinical practice. By synchronising clinical and research visits, within an excellent NIHR BRC facility, the patient journey is not excessively burdened. Data capture absorbs a wealth of information from the comprehensive characterisation process undertaken in the clinic.

Where WATCH is aligned to a participant’s parallel clinical care needs, there is a potential susceptibility to missing data. Unlike formal research visits, which are rigorously protocol driven, clinical care is rarely so. There is thus a theoretical risk that WATCH data capture may be more prone to missing data. Whilst some data are incomplete, Table 6 demonstrates the high levels of data acquisition achieved by the WATCH study.

A core goal of the WATCH study is to build upon notable recent studies (SARP, U-BIOPRED and the BTS Difficult Asthma Network) in understanding the basis and clinical heterogeneity of asthma [23, 26, 31]. It is important to recognise distinctions between those previously studied populations and that of WATCH. Firstly, those prior studies were multi-centred, encompassing a wide range of study sites, whereas WATCH has been initially established over just two sites. As a formally commissioned Regional Centre for Severe Asthma, however, the WATCH cohort is composed of patients from across South Central England. Like the multi-centre cohorts, WATCH patients come from a variety of environments (inner city urban to rural farming) and span a representative social spectrum.

Another key distinction is that the WATCH cohort describes patients with difficult asthma, defined as problematic asthma despite treatment with high dose therapies, or continuous or frequent use of oral steroids. This does *not* exclude subjects where poor asthma control may be related to non-adherence to therapy or poorly controlled co-morbidities. Where such forerunner studies have looked exclusively at Severe Asthma, variably defined over the past few decades [32], a proportion of the difficult to manage patients have been neglected despite their significant morbidity [2]. Similarly, patients have been excluded by stringent entry criteria, such as the demonstration of bronchodilator reversibility or positive methacholine challenge to confirm an asthma diagnosis (SARP and U-BIOPRED) [33] or exclusion of patients with a significant smoking history (SARP) [23]. The broad inclusion criteria employed by the WATCH study might therefore be criticised for diluting its severe asthma population, but, as per our primary objective, it accurately reflects the true clinical spectrum of high morbidity patients who frequently challenge day-to-day clinical practice in a Regional Difficult Asthma Service.

One of the specifications for a Regional Asthma Centre is the long-term follow up care of patients [7]. As patients return for regular follow up appointments, prospective data capture is easily facilitated. Preceding severe asthma cohort studies have largely focused on cross-sectional perspectives. The BTS Severe Asthma Registry identified 5 clusters (*n* = 349) but found stability of cluster membership to be just 52% after 3 years [34]. Coupled with recognition of temporal variability in blood and sputum inflammometry [12, 35], the need for better longitudinal perspectives of difficult asthma is now of critical interest [36]. This is possible within the WATCH study, which also collects a biobank repository to support deeper phenotyping analysis, such as that performed in U-BIOPRED and the ADEPT study [37–39].

The breadth of data captured over time by the WATCH study requires careful data management. Data is collected in a bespoke electronic database with multiple exclusive functions. It can support patient specific follow up CRFs to guide individualised data capture (Additional file 2) and give snapshots of data acquisition. By directly interfacing with the hospital electronic systems it can also retrieve the results of clinical investigations over a 10-year period. Such data extraction can aid longitudinal phenotypic characterisation by easily incorporating a temporal assessment for parameters, such as blood eosinophil count, serum total IgE and fungal specific IgE. The database can also easily identify nested cohorts of patients based on any number of clinical parameters, such as lung function, treatment or investigation results. Finally, the database can be interrogated to produce datasets specific to hypotheses of interest.

Supported by this database and biobank, it is envisaged that the “parent” WATCH cohort of difficult asthma patients would act as a platform from which “offspring” studies can be readily developed and undertaken. At the time of writing, the parent WATCH study is hosting several offspring studies covering a diversity of research interests. These include observational studies of mindfulness therapies and breathing pattern disorders as well as industry and institutional basic science collaborations, including an NIH funded Epigenetics of Severe Asthma study in collaboration with the La Jolla Institute of Allergy and Immunology, San Diego, USA. These collaborations have generated significant institutional research income; £450,000 from the pharmaceutical industry and £400,000 from the NIH, which contribute to the maintenance and logistics of the parent WATCH study. From a patient perspective, offspring studies offer further clinical characterisation (such as through bronchoscopy) and/or access to novel therapies (such as mindfulness). Collaborations are therefore welcomed but require a feasibility assessment by a WATCH Research Approvals Committee primarily to prevent overburdening the cohort.

The advantages, simplicity and potential of the WATCH study belie the logistic complexity involved in establishing and maintaining a longitudinal study with unlimited ongoing enrolment that is embedded in clinical practice. Funding was obtained through collaborations with industry and charitable organisations, whilst the well-established Clinical Research Facility at UHSFT provided infrastructure support. Meticulous integration and co-ordination of clinical and research resources have been critical in harnessing the full potential of the WATCH study. Though challenging, this unique and pragmatic research model could potentially be reproduced in other NHS institutions that provide specialist clinical services.

Lastly, the WATCH study is mindful of the national and international efforts to bring together data from multiple centres in order to enhance the power of studies, be they clinical observational, mechanistic, or intervention studies. The study will therefore be aligning itself to the UK Severe Asthma Registry (previously the BTS Severe Asthma Registry) and the European Respiratory Society Clinical Research Collaboration on Severe Asthma. To this end, the Severe Heterogeneous Asthma Research collaboration, Patient-centred (SHARP) was formed following an Asthma UK-led pan-European project, which assesses the needs in severe asthma [40, 41]. Through a judicious process of harmonisation of protocols and variables collected, this will provide unprecedented data on large cohorts, thus improving understanding of disease mechanisms, sharing experiences and enabling improved asthma care across Europe through more targeted, stratified-medicine, therapeutic approaches.

In summary, the WATCH study aims to create a unique clinical-research collaboration that engages patients in meaningful research alongside their routine clinical care. It relies on a comprehensive clinical assessment process that can be accessed in pragmatic fashion to satisfy research needs. That pragmatic approach will inevitably impose some limitations on data acquisition. Nevertheless the WATCH study promises to yield a highly informative real-life longitudinal perspective on difficult asthma and serve as a reliable vehicle for a considerable body of future work.

## Additional files

Additional file 1: WATCH enrolment CRF. (PDF 200 kb)

Additional file 2: WATCH follow-up CRF. (PDF 312 kb)

Additional file 3: Appendix A Clinical Investigations. Appendix B Lung Function Test. Appendix C Biobank Samples. (DOCX 25 kb)

## Data Availability

The datasets used and/or analysed during the current study are available from the corresponding author on reasonable request. All requests for datasets are reviewed for approval by the WATCH Research Approvals Committee.

## Abbreviations

A1AT: Alpha-1-Antitrypsin Level
ACQ-6: 6 point Asthma Control Questionnaire
ACQ-7: 7 point Asthma Control Questionnaire
ACS: Attention Control Scale
ANA: Antinuclear Antibody
ANCA: Anti-Neutrophil Cytoplasmic Antibody
ARTP: Association of Respiratory Technology and Physiology
BAL: Bronchoalveolar lavage
BIA: Bioelectrical Impedance Analysis
BTS: British Thoracic Society
CNAQ: Council on Nutritional Appetite Questionnaire
COPD: Chronic Obstructive Pulmonary Disease
CRF: Case Report Form
CT: Computed Tomography
DEXA: Dual Energy X-Ray Absorptiometry
ENT: Ear Nose & Throat
EQ-5D-5 L: 5 level version of the EQ-5D Questionnaire developed by the EuroQol Group
ERS: European Respiratory Society
FBC: Full Blood Count
FeNO: Fractional Exhaled Nitric Oxide, measured at a flow rate of 50 ml/s
GI: GastroIntestinal
HADS: Hospital Anxiety and Depression Score
HRCT: High Resolution Computed Tomography
IgA: Immunoglobulin A
IgE: Immunoglobulin E
IgG: Immunoglobulin G
IgM: Immunoglobulin M
MAAS: Mindful Attention Awareness Scale
MARS-A: Medication Adherence Report Scale for Asthma
MDT: Multidisciplinary Team
MUST: Malnutrition Universal Screening Test
NHLBI: National Heart, Lung and Blood Institute
NHS: National Health Service
NHSE: National Health Service England
NIH: National Institutes of Health
OGD: Oesophagogastroduodenoscopy
PTH: Parathyroid Hormone
SARP: Severe Asthma Research Project
SGRQ: St George’s Respiratory Questionnaire
SHARP: Severe Heterogeneous Asthma Research collaboration, Patient-centred
SNOT-22: 22 point Sino-Nasal Outcome Test
SOP: Standard Operating Procedure
T4: Free Thyroxine
TSH: Thyroid Stimulating Hormone
U-BIOPRED: Unbiased Biomarkers in Prediction of Respiratory Disease Outcomes
UHSFT: University Hospital Southampton Foundation Trust
UK: United Kingdom
WATCH: Wessex Asthma Cohort of Difficult Asthma
