# Supplementary material for: Protocol for the Wessex AsThma CoHort of difficult asthma (WATCH): a pragmatic real-life longitudinal study of difficult asthma in the clinic
Source: BMC Pulm Med. 2019 May 24;19:99. doi: 10.1186/s12890-019-0862-2 (PMC6534885; doi:10.1186/s12890-019-0862-2)
Supplement: Supplementary file 1 — WATCH enrolment CRF. (PDF 200 kb) [file 12890_2019_862_MOESM1_ESM.pdf]

W A T C H
Wessex Asthma Lifetime Cohort
Ethics REC No.: 14/WM/1226

Enrolment CRF

Subject No. W 0 0 _ _ _ / _ _ _ **Visit Date** D D / M M / Y Y

1. Screening and Consent

1.1 Screening Date D D / M M / Y Y

1.2 Study Site Southampton O Isle of Wight O Portsmouth O

1.3 Is a new patient? Yes O No O
(i.e. new to asthma clinic)

1.4 Inclusion Criteria

All patients referred to the Adult or Transitional Regional Asthma Clinic at UHSFT or other participating hospitals who are on high-dose therapies (high dose ICS plus multiple controller therapies), with or without continuous or frequent oral steroids (or oral steroid sparing strategies/ biological therapies) as defined by the British Thoracic Society (BTS) Adult Asthma Management Guidelines (2016).

Does the patient fulfill these criteria? ☐

1.5 Informed consent

Has the patient been informed both verbally and in writing about the objectives of the cohort, the methods, the benefits and potential risks and of the discomfort to which he/she may be exposed and has given written consent to participate in the study prior to any study specific procedures being carried out?

Yes O No O → *Obtain informed consent or reschedule visit*

→ **Consent taken by** __________

Date of consent D D / M M / Y Y

Consent form version __________

Copies to: **a) Patient?** ☐ **b) Hospital Notes?** ☐ **c) Site file? (original)** ☐

2. Demographic Data

2.1 Date of birth D D / M M / Y Y **Age** |__|__| years

2.2 Gender Male O Female O

2.3 Ethnic Group
Tick ONE only

**Caucasian**	O	**Polynesian**	O
**Afro-Caribbean**	O	**Indian Sub-continent**	O
**Hispanic**	O	**Mixed**	O → **Specify**
**North-east Asian**	O	**Other**	O → **Specify**
**South-east Asian**	O		

Comments ....

2.4 Height |__|.|__|__| metres **How obtained?** Measured O Asked patient O

2.5 Weight |__|__|.|__|__| kg **How obtained?** Measured O Asked patient O

2.6 Working Status
Tick ONE only

**Working full-time**	O
**Working part-time due to asthma-related ill-health**	O
**Working part-time due to other causes**	O
**Not working due to asthma-related ill-health**	O
**Not working due to other causes**	O → **Other causes**

2.7 Occupation |______|
(Include position and nature of work)

2.8 Has there ever been an occupational component to your asthma? Yes O No O Not known O

→ **Note any known irritants / products / components or stressors associated**
....

2.9 Do you have regular exposure to pets / animals?

a) In your household? **Yes** O **No** O
→ **Specify:** ....

b) Elsewhere? **Yes** O **No** O
→ **Specify:** ....

3. Asthma History

3.1 Year of diagnosis Y Y Y Y **or Not known** ☐

3.2 Age at diagnosis **Under 6** ☐ **6 to 11** ☐ **12 to 18** ☐ **19 to 40** ☐ **41 to 60** ☐ **Over 60** ☐ **N/K** ☐

3.3 Year of onset of symptoms Y Y Y Y **or Not known** ☐

3.4 Asthma triggers *(Tick all that apply)*

**Cold air**		☐
**Climate**	e.g. changes in temperature or humidity	☐
**Fumes**	e.g. smoke, perfume or sprays	☐
**Allergens**	e.g. HDM, animals or moulds	☐
**Medications**	e.g. Beta-blockers or NSAIDs	☐
**Emotion**	e.g. stress or bereavement	☐
**Hormonal**	e.g. pre-menstrual or during pregnancy	☐
**Night time or early morning**		☐
**Foods**	e.g. tartrazine, MSG, sulphites, peanuts or shellfish	☐
**Workplace**	e.g. Exposure to agents you have become sensitised to	☐
**Viral respiratory tract infections, common cold or influenza**		☐
**Exercise**		☐
**Air pollution**		☐
**Alcohol**		☐
**Others**		☐ → **Specify**

3.5 Do you self-manage exacerbations? Yes O No O

3.6 Total number of asthma-related Intensive Care Unit (ICU) visits (ever) |__|__| **or Not known** ☐

3.7 Ever intubated? Yes O No O Not known O

3.8 In the last 12 months (approx.), how many asthma-related... *(If zero, enter zero)*

a) ... visits to GP |__|__| **or Not known** ☐

b) ... visits to Emergency Dept. (ED) |__|__| **or Not known** ☐

c) ... hospitalisations |__|__| **or Not known** ☐

d) ... courses of oral corticosteroids (OCS) |__|__| **or Not known** ☐
(If on maintenance, record the number of times needed to double-dose)
→ **If > 0: Dose** |____| *mg/day* **Duration** |____| *days*

e) ... days lost from work/education |__|__| **or Not known** ☐ **or Not applic.** ☐ *(i.e. not in work nor education)*

f) ... courses of antibiotics |__|__| **or Not known** ☐
→ **If > 0, which antibiotics?**

3.9 Are you on long-term antibiotics? Yes O No O
→ **Which antibiotic(s)?**

3.10 Any family history of asthma? **Yes** O **No** O **Not known** O
→ **Who?** *(Tick all that apply)* **Father** ☐ **Mother** ☐ **Brother** ☐ **Sister** ☐ **Children** ☐ **Other** ☐ → **Specify:**

3.11 Any deaths in the family due to asthma? **Yes** O **No** O **Not known** O

4. Medications

4.1 Please record all 'baseline' asthma AND NON-ASTHMA medications ongoing as at the start of this visit.

NB Remember to include prn medications, including e.g. salbutamol, epipen and antihistamine

	Drug	Dose		Frequency	Route	Started		
		Quantity	Units			Month	Year	NK
1.						MM /	YY	☐
2.						MM /	YY	☐
3.						MM /	YY	☐
4.						MM /	YY	☐
5.						MM /	YY	☐
6.						MM /	YY	☐
7.						MM /	YY	☐
8.						MM /	YY	☐
9.						MM /	YY	☐
10.						MM /	YY	☐
11.						MM /	YY	☐
12.						MM /	YY	☐
13.						MM /	YY	☐
14.						MM /	YY	☐
15.						MM /	YY	☐
16.						MM /	YY	☐
17.						MM /	YY	☐
18.						MM /	YY	☐
19.						MM /	YY	☐
20.						MM /	YY	☐
21.						MM /	YY	☐
22.						MM /	YY	☐
23.						MM /	YY	☐
24.						MM /	YY	☐
25.						MM /	YY	☐

Page completed by ____________

Date completed DD / MM / YY

5. Asthma Treatment Details

Have you ever been treated with ...

5.1 Omalizumab (Xolair)? Yes O No O
→ *Please enter details of dates, dose etc into Section 4 'Medications' above*

5.2 Mepolizumab? Yes O No O
→ *Please enter details of dates, dose etc into Section 4 'Medications' above*

5.3 Reslizumab? Yes O No O
→ *Please enter details of dates, dose etc into Section 4 'Medications' above*

		Start Date	Ongoing?	End Date
**5.4 Bronchial Thermoplasty?**	Yes O →	D D / M M / Y Y	Yes O	
	No O →	*Q5.5*	No O →	D D / M M / Y Y

5.5 Thermal laminar Airflow Device? Yes O → D D / M M / Y Y
No O → *Q5.6*

e.g. from LASER study

5.6 Inhaler technique: **Date technique assessed** D D / M M / Y Y

Technique:
- **Good** O — *No changes needed*
- **Sub-optimal** O — *Minor changes needed*
- **Poor** O — *Major changes, or change of inhaler needed*
- **Not done** O

5.7 Action plan:
Adequate plan pre-existing? Yes O No O
Action plan given? Yes O No O
→ **Date given** D D / M M / Y Y

Comments ....

....

Page completed by ____________

Date completed D D / M M / Y Y

6. Smoking History

6.1 Smoking status

Never smoked	O	→ *Q6.3*		
Current smoker	O	→ **Age started** ⌊__⌋ years		
Ex-smoker	O	→ **Age started** ⌊__⌋ years	**Age stopped**	⌊__⌋ years

6.2 Pack-year history

a) Cigarettes
- **Number smoked per day** ⌊___⌋ cigarettes
- **Number of years smoked** ⌊__⌋ years

b) Pipes / Cigars
- **Number smoked per day** ⌊__⌋ pipes / cigars → **Pipes** O / **Cigars** O / **Other** O → **Specify** ⌊____⌋
- **Number of years smoked** ⌊__⌋ years

c) Cigarillos
- **Number smoked per day** ⌊__⌋ cigarillos
- **Number of years smoked** ⌊__⌋ years

d) Tobacco
- **Grams smoked per day** ⌊__⌋.⌊_⌋ gms
- **or**
- **Ounces smoked per day** ⌊__⌋.⌊_⌋ oz
- **Number of years smoked** ⌊__⌋ years

Pack-years Calculation

= Cigarettes per day x years smoked / 20

1 Cigar/pipe/cigarillo = 5 cigarettes

12.5g or 0.5oz tobacco = 20 cigarettes

Total pack years ⌊____⌋

6.3 Passive exposure status

- **Currently** O
- **In the past** O
- **Never** O

→ **From:** **Parents?** ☐ **Spouse?** ☐ **Others?** ☐ → **Specify** ⌊____⌋

Tick all that apply

Number of years ⌊__⌋ years

E-cigarettes

6.4 Uses e-cigarettes regularly? Yes O No O → *Next Section*

→ **6.4.1 Used for how long?** ⌊__⌋ years ⌊__⌋ months

6.4.2 Nicotine dose ⌊__⌋ mgs *(Common values are 0, 6, 12, 18, 24 and 36)*

6.4.3 How long does it take to use 10ml of e-liquid? ⌊__⌋ weeks ⌊_⌋ days

6.4.4 Which flavour(s)? ⌊________⌋

Comments ....

....

....

Page completed by ⌊____⌋

Date completed D D / M M / Y Y

7. Atopy History

7.1 Do you have any history of FOOD allergies?

Yes O No O → *Q7.2* Not known O

→ **Please list the foods and the symptoms they cause** *(Tick all that apply)*

	Food	Respiratory	Ocular	Nasal	Rash	Vomiting	Anaphylaxis	Other	If 'Other', specify
a)		☐	☐	☐	☐	☐	☐	☐	
b)		☐	☐	☐	☐	☐	☐	☐	
c)		☐	☐	☐	☐	☐	☐	☐	
d)		☐	☐	☐	☐	☐	☐	☐	
e)		☐	☐	☐	☐	☐	☐	☐	
f)		☐	☐	☐	☐	☐	☐	☐	

7.2 Do you have any history of DRUG allergies?

Yes O No O → *Q7.3* Not known O

→ **Please list the drugs and the symptoms they cause** *(Include NSAID intolerance)*

	Drug	Respiratory	Ocular	Nasal	Rash	Vomiting	Anaphylaxis	Other	If 'Other', specify
a)		☐	☐	☐	☐	☐	☐	☐	
b)		☐	☐	☐	☐	☐	☐	☐	
c)		☐	☐	☐	☐	☐	☐	☐	
d)		☐	☐	☐	☐	☐	☐	☐	
e)		☐	☐	☐	☐	☐	☐	☐	
f)		☐	☐	☐	☐	☐	☐	☐	

7.3 Do you have any family history of atopy? Yes O No O Not known O

(e.g. asthma, food allergy, hay fever or eczema)

→ **Who?** *(Tick all that apply)*

Father ☐ Mother ☐

Brother ☐ Sister ☐ Children ☐

Other ☐ → **Specify:** ____________

Comments ....

....

....

Page completed by ____________

Date completed D D / M M / Y Y

8. Nasal Disease

8.1 Do you have any history of rhinitis?	Yes O	No O	Not known O
8.2 Do you have any history of nasal polyps?	Yes O	No O	Not known O
8.3 Have you had nasal surgery?	Yes O	No O	Not known O

Comments ....

....

8.4 Have you any history of Perennial Allergy?	Yes O	No O	Not known O
8.5 Have you any history of Seasonal Allergy?	Yes O	No O	Not known O

Comments ....

....

8.6 CT Sinuses performed? Yes O No O N/K O
(Clinical indication only)

→ Date performed D D / M M / Y Y

Findings:
- ☐ Normal
- ☐ Polyps
- ☐ Mucosal thickening
- ☐ Sinus opacification / abnormality
- ☐ Abnormal nasal turbinates
- ☐ Other → Specify [____________]

9. Reflux History

9.1 Diagnosed with GORD? Yes O No O N/K O
(pt self-report or clinician diagnosis in letter/notes)

9.2 Any symptoms of GORD?
- Yes, and ongoing despite on treatment O
- Yes, but not on treatment at present O
- No, on treatment and symptoms controlled O
- No O

9.3 Oesophageal testing performed / planned?
(Only if clinically indicated)

	Yes	No / Never	Requested, not yet done	Requested, but not done for some reason e.g. DNA
a) Barium swallow?	O → Date D D / M M / Y Y	O	O	O
b) OGD?	O → Date D D / M M / Y Y	O	O	O
c) Oesophageal manometry PH / impedance?	O → Date D D / M M / Y Y	O	O	O
d) Other? → Specify [____________]	O → Date D D / M M / Y Y	O	O	O

10. Psychological History

10.1 Diagnosis* of:

- **Depression?**	Yes O	No O
- **Anxiety?**	Yes O	No O
- **Other psychological / psychiatric comorbidity?**	Yes O	No O

→ **Specify** [ ]

** Patient self-report or clinician diagnosis in letter / notes.*

10.2 Seen by mental health professionals?

	Yes	No / Never	Requested, not yet done	Requested, but not done for some reason e.g. DNA
**a) Psychiatrist?**	O → **Date** D D / M M / Y Y; Completed O; Ongoing O	O	O	O
**b) Psychologist?**	O → **Date** D D / M M / Y Y; Completed O; Ongoing O	O	O	O
**c) Specialist asthma psychologist?**	O → **Date** D D / M M / Y Y; Completed O; Ongoing O	O	O	O
**d) Other?**	O → **Date** D D / M M / Y Y; Completed O; Ongoing O	O	O	O

→ **Specify** [ ]

Page completed by [ ]

Date completed D D / M M / Y Y

11. Breathing Pattern Disorders

11.1 Diagnosis* of:

- **Vocal cord dysfunction?**	Yes O	No O
- **Dysfunctional breathing?**	Yes O	No O

** patient self-report or clinician diagnosis in letter / notes.*

11.2 Investigations for VCD

	Yes	No / Never	Requested, not yet done	Requested, but not done for some reason e.g. DNA
**a) Nasendoscopy?**	O	O	O	O
→ **Date** D D / M M / Y Y				
**b) Other?**	O	O	O	O
→ **Date** D D / M M / Y Y				
→ **Specify** ________				

11.3 Seen by BPD/VCD specialist?

	Yes	No / Never	Requested, not yet done	Requested, but not done for some reason e.g. DNA
**a) Physiotherapist?**	O<br>→ **Date** D D / M M / Y Y<br>Completed O<br>Ongoing O	O	O	O
**b) Specialist asthma physiotherapist?**	O<br>→ **Date** D D / M M / Y Y<br>Completed O<br>Ongoing O	O	O	O
**c) Speech therapist?**	O<br>→ **Date** D D / M M / Y Y<br>Completed O<br>Ongoing O	O	O	O
**d) Other?**	O<br>→ **Date** D D / M M / Y Y<br>Completed O<br>Ongoing O	O	O	O

→ **Specify** ________

12. Comorbidities and Procedures

12.1 Comorbidities

Condition	Previously had? Yes	No	N/K	Currently have? Yes	No	N/K
a) COPD	O	O	O	O	O	O
b) Allergic Bronchopulmonary Aspergillosis or SAFS	O	O	O	O	O	O
c) Non-CF Bronchiectasis	O	O	O	O	O	O
d) Cystic Fibrosis	O	O	O	O	O	O
e) Bronchiolitis	O	O	O	O	O	O
f) Churg Strauss Syndrome	O	O	O	O	O	O
g) Chronic urticaria / angioedema	O	O	O	O	O	O
h) Sulphite sensitivity	O	O	O	O	O	O
i) Salicylate (aspirin) sensitivity	O	O	O	O	O	O
j) Eczema	O	O	O	O	O	O
k) Latex allergy	O	O	O	O	O	O
l) Sleep apnoea	O	O	O	O	O	O
**Other Conditions** *(write in)*						
1. ________	O			O	O	O
2. ________	O			O	O	O
3. ________	O			O	O	O
4. ________	O			O	O	O
5. ________	O			O	O	O
6. ________	O			O	O	O

12.2 Procedures

	Ever Received? Yes	No	N/K	If 'Yes', Date of Procedure
a) Bariatric surgery	O	O	O	D D / M M / Y Y
b) Hiatus hernia repair	O	O	O	D D / M M / Y Y
c) Hysterectomy	O	O	O	D D / M M / Y Y
**Other Procedures** *(write in)*				
1. ________	O			D D / M M / Y Y
2. ________	O			D D / M M / Y Y
3. ________	O			D D / M M / Y Y
4. ________	O			D D / M M / Y Y
5. ________	O			D D / M M / Y Y
6. ________	O			D D / M M / Y Y

13. Imaging - HRCT

13.1 HRCT performed? Yes O No O

13.2 Date performed D D / M M / Y Y

13.3 HRCT evidence of:

	Yes	No
Central bronchiectasis	O	O
Other bronchiectasis	O	O
Bronchial dilation without bronchiectasis	O	O
Bronchial wall thickening	O	O
Ground glass shadowing	O	O
Mucus plugging	O	O
Air trapping	O	O
Emphysema - Centrilobular	O	O
Emphysema - Panacinar	O	O
Other category / other findings	O	O

Specify in 'Comments' below

or

Scan reported as normal by radiologist	O	O

Comments ....

14. Imaging - DEXA

14.1 DEXA scan performed? Yes O No O

14.2 Date performed D D / M M / Y Y

14.3 Spinal bone density (L1-4) T score |__________|

14.4 Femoral neck bone density (L1-4) T score: a) Left |__________|

b) Right |__________|

Comments ....

15. Concordance

15.1 % concordance preventer |__|__|__| %

15.2 Number of refills for the reliever |__|__| **per how many weeks?** |__|__| *wks*

15.3 Clinical impression of concordance

- Good O
- Sub-optimal O
- Poor O
- Unable to comment O

Comments ....

At this point ask the patient to complete the Questionnaires.

Page completed by |__________|

Date completed D D / M M / Y Y

16. Dietary Assessment

16.1 Body composition assessed? Yes O No O → *Q16.2*

**Test**	**Result**	
FFM		kg
FM		kg
FFMi		kg/m2
SMM		kg
PhA		degrees

16.2 Seen by asthma specialist dietician?

Tick ONE only

- **Not required** O
- **Ongoing** O
- **To be seen** O
- **Discharged, treated** O
- **Discharged, DNA** O

17. Research Samples

17.1 Date research bloods taken D D / M M / Y Y

17.2 Date urine taken D D / M M / Y Y

17.3 Date sputum taken D D / M M / Y Y

17b. Research Samples for Patients Enrolled in "WATCH NIH" Study

17b.1 Date PBMC #1 D D / M M / Y Y

17b.2 Date PBMC #2 D D / M M / Y Y

17b.3 Date PBMC #3 D D / M M / Y Y

17b.4 Date Sputum D D / M M / Y Y

17b.5 Date Bronch D D / M M / Y Y

18. FeNO (Exhaled Nitric Oxide)

18.1 Was FeNO(50) done? Yes O No O → *18.2*

→ **18.1.1 Date performed** D D / M M / Y Y

18.1.2 Where done? **UHS PFT** O **UHS Research Lab.** O **IOW OPD Clinic** O **UHS Resp. Centre** O **Portsmouth OPD Clinic** O

18.1.3 Did the patient refrain from eating and drinking for at least 2 hours prior to the FeNO measurement? **Yes** O **No** O → *Consider performing test at next visit* **Unknown** O

18.1.4 Bedfont (50ml/sec) **1.** ______ **2.** ______ **3.** ______

18.2 Was Multiple Flow NO done? Yes O No O → *Next Section*

(University Hospital Southampton only)

→ **18.2.1 Date performed** D D / M M / Y Y

18.2.2 Test results *Attach the results printout to this CRF.*
Please ensure the Subject No. is written clearly on the printout.

Comments ....

....

....

19. Spirometry

19.1 Was Spirometry done? Yes O → *19.2* No O → *Next Section*

Use historical REVERSIBILITY data if available within last 12 months

19.2 Date of test D D / M M / Y Y

19.3 Where done? UHS PFT O UHS Research Lab. O IOW OPD Clinic O UHS Resp. Centre O Portsmouth OPD Clinic O

19.4 Was acceptable spirometry obtained? Yes O No O
(Only record parameters of good quality)

19.5 Was a washout period observed? Yes O No O → *Record results as POST-BD*
(SABA 4 hours and LABA 12 hours)

19.6 Last use of short-acting β-agonist Date D D / M M / Y Y Time H H : M M or NA ☐ *e.g. if using maintenance inhaler as reliever)*
(Salbutamol, Ventolin, Terbutaline, Bricanyl)

19.7 Last use of long-acting β-agonist Date D D / M M / Y Y Time H H : M M
(Formoterol, Fumarate, Salmeterol, Bambuterol)

19.8 Test results *Attach the results printout to this CRF.*
Please ensure the Subject No. is written clearly on the printout.

Comments ....

....

....

20. Lung Volumes

20.1 Date of test D D / M M / Y Y

20.2 Where done? UHS PFT O UHS Research Lab. O IOW OPD Clinic O Portsmouth OPD Clinic O

20.3 Method used Plethysmography O Nitrogen washout O Other O → **Specify** ____________

20.4 Test results *Attach the results printout to this CRF.*
Please ensure the Subject No. is written clearly on the printout.

Comments ....

....

....

21. Single Breath Diffusion / Transfer Facto

21.1 Was test done? Yes O No O → *Next Section*

→ **21.2 Date of test** D D / M M / Y Y

21.3 Where done? UHS PFT O UHS Research Lab. O IOW OPD Clinic O Portsmouth OPD Clinic O

21.4 Test results *Attach the results printout to this CRF.*
Please ensure the Subject No. is written clearly on the printout.

Comments ....

....

....

22. Impulse Oscillometry (IOS)

22.1 Was test done? Yes O No O → *Next Section*

If Yes:

22.2 Date of tests D D / M M / Y Y

22.3 Where done? **UHS PFT** O **UHS Research Lab.** O **IOW OPD Clinic** O **Portsmouth OPD Clinic** O

22.4 Was a washout period observed? *(SABA 4 hours and LABA 12 hours)* **Yes** O **No** O → *Record results as POST-BD*

22.5 Last use of short-acting β-agonist *(Salbutamol, Ventolin, Terbutaline, Bricanyl)* **Date** D D / M M / Y Y **Time** H H : M M **or NA** ☐ *e.g. if using maintenance inhaler as reliever)*

22.6 Last use of long-acting β-agonist *(Formoterol, Fumarate, Salmeterol, Bambuterol)* **Date** D D / M M / Y Y **Time** H H : M M

22.7 Test results *Attach the results printout to this CRF.*
Please ensure the Subject No. is written clearly on the printout.

Comments ....

....

....

23. Multiple Breath Nitrogen Washout (MBNW) *(University Hospital Southampton only)*

23.1 Was test done? Yes O No O → *Next Section*

→ **23.2 Date of test** D D / M M / Y Y

23.3 Time of test H H : M M

23.4 Test Results *Attach the results printout to this CRF.*
Please ensure the Subject No. is written clearly on the printout.

Comments ....

....

....

Page completed by |________|

Date completed D D / M M / Y Y

24. Allergy Testing

24.1 Skin prick test form attached? Yes O No O

24.2 Date performed D D / M M / Y Y

24.3 Number of days H1 or H2 anti-histamines omitted? n/a ☐ or ⌊__⌋ days
(If greater than 3 days just enter '3')

24.4 Number of days Tricyclic Antidepressants (TCAs) omitted? n/a ☐ or ⌊__⌋ days
(If greater than 7 days just enter '7')

Comments ....

....

....

25. Allergy RAST Testing

	Allergen	Test Date	Result	Grade	Notes
1.		D D / M M / Y Y			
2.		D D / M M / Y Y			
3.		D D / M M / Y Y			
4.		D D / M M / Y Y			
5.		D D / M M / Y Y			

26. Sputum

26.1 Sputum eosinophils ⌊__|__|__⌋ %

26.2 Sputum neutrophils ⌊__|__|__⌋ %

27. Bronchoscopy

27.1 Bronchoscopy performed? Yes O No O
(Only if clinically indicated)

27.2 Date performed D D / M M / Y Y

27.3 Histological findings? Yes O No O
(Attach printout report)

27.4 Brushings and Biopsies

	Number	Time	Site of Samples
**a) Biopsies**		H H : M M	
**b) Brushings**		H H : M M	

27.5 Bronchial lavage

In ⌊__|__⌋ ml **Out** ⌊__|__⌋ ml

Comments ....

....

....

Page completed by ⌊________⌋

Date completed D D / M M / Y Y

28. Other Research Study Participations

- a) WATCH BI ☐
- b) WATCH NIH ☐
- c) WATCH GSK ☐
- d) Panos ☐
- e) WSAC ☐
- f) UBIOPRED ☐
- g) SoMOSA ☐
- h) RASP UK ☐
- i) RASP Bronch ☐
- j) MEPO ☐
- k) MIDAS ☐

Others (specify)

- i ____________ ☐
- ii ____________ ☐
- iii ____________ ☐
- iv ____________ ☐

Page completed by ____________

Date completed D D / M M / Y Y
