# Supplementary material for: Protocol for the Wessex AsThma CoHort of difficult asthma (WATCH): a pragmatic real-life longitudinal study of difficult asthma in the clinic
Source: BMC Pulm Med. 2019 May 24;19:99. doi: 10.1186/s12890-019-0862-2 (PMC6534885; doi:10.1186/s12890-019-0862-2)
Supplement: Supplementary file 2 — WATCH follow-up CRF. (PDF 312 kb) [file 12890_2019_862_MOESM2_ESM.pdf]

W A T C H

Wessex Asthma Lifetime Cohort

Ethics REC No.: 14/WM/1226

University Hospital Southampton NHS Foundation Trust

Year 1 Follow-up CRF

Study Site: AAAA

Subject No. W00999/XXX

Visit Date D D / M M / Y Y

1. Re-consent

Last consent: 21 Mar 2017 (Enrolment CRF)

1.1 Re-consent

Ongoing verbal consent obtained? **Yes** O **No** O

Re-consent taken by __________

Enrolment written consent: 21 Mar 2017

2. Asthma History

Responses from 21 Mar 2017 (Enrolment CRF)

Question	Response	Responses from 21 Mar 2017 (Enrolment CRF)
**2.1 Do you self-manage exacerbations?**	Yes O No O	*Yes*
**2.2 Since your last WATCH study visit, how many asthma-related Intensive Care Unit (ICU) visits?**	___ **or Not known** ☐	*0 visits*
**2.3 Since your last WATCH study visit, have you been intubated?**	Yes O<br>No O<br>Not known O	*No*
**2.4 Since your last WATCH study visit, how many asthma-related...**	*(If zero, enter zero)*	
**a) ... visits to GP**	___ **or Not known** ☐	*2 GP visits*
**b) ... visits to Emergency Dept. (ED)**	___ **or Not known** ☐	*0 ED visits*
**c) ... hospitalisations**	___ **or Not known** ☐	*0 hospitalisations*
**d) ... courses of oral corticosteroids (OCS)**<br>(If on maintenance, record the number of times needed to double-dose)	___ **or Not known** ☐	*6 OCS courses*
	**If > 0: Dose** ___ *mg/day*	*Missing*
	**Duration** ___ *days*	*Missing*
**e) ... days lost from work/education**	___ **or Not known** ☐<br>**or Not applic.** ☐ *(i.e. not in work nor education)*	*0 days lost*
**f) ... courses of antibiotics**	___ **or Not known** ☐<br>**If > 0, which antibiotics?** __________<br>__________	*6 courses amoxillin, doxycycline*
**2.5 Are you on long-term antibiotics?**	Yes O No O<br>**Which antibiotic(s)?** __________<br>__________	*No*

Page completed by __________

Date completed D D / M M / Y Y

3. Medications

3.1 Please document any changes to the following medications which were 'Ongoing' at the last visit.

(Either tick the 'Still Ongoing?' check boxes or, if ended, record when.)

					Started			Still	Ended		
**Drug**	**Quantity**	**Units**	**Frequency**	**Route**	**Month**	**Year**	**NK**	**Onging?**	**Month**	**Year**	**NK**
Fostair	100/6	mcg	2 puffs BD	Inhaler	1	2015	☐	☐			☐
Ciclesonide	80	mcg	2 puffs OD	Inhaler	1	2015	☐	☐			☐
Spiriva	2.5	mcg	2 puffs OD	Inhaler	1	2015	☐	☐			☐
Ventolin	100	mg	PRN	Inhaler	1	2015	☐	☐			☐
Montelukast	10	mg	nocte	PO	1	2015	☐	☐			☐
Sertraline	50	mg	OD	PO	5	2016	☐	☐			☐
Alendronic Acid	70	mg	once a week	PO	11	2016	☐	☐			☐
Lansoprazole	30	mg	OD	PO			☐	☐			☐

3.2 New asthma and non-asthma medication, and any changes in doses to existing mediation, since last visit.

		Dose				Started				Ended		
	**Drug**	**Quantity**	**Units**	**Frequency**	**Route**	**Month**	**Year**	**NK**	**Onging?**	**Month**	**Year**	**NK**
1.								☐	☐			☐
2.								☐	☐			☐
3.								☐	☐			☐
4.								☐	☐			☐
5.								☐	☐			☐
6.								☐	☐			☐
7.								☐	☐			☐
8.								☐	☐			☐
9.								☐	☐			☐
10.								☐	☐			☐

Page completed by ____________

Date completed D D / M M / Y Y

4. Asthma Treatment Details

Since the last WATCH Visit have you been treated with ...

Responses from 21Mar 2017 (Enrolment CRF)

4.1 Omalizumab (Xolair)? Yes O No O
→ *Please enter details into Section 3 'Medications' above* — *No*

4.2 Mepolizumab? Yes O No O
→ *Please enter details into Section 3 'Medications' above* — *No*

4.3 Reslizumab? Yes O No O
→ *Please enter details into Section 3 'Medications' above* — *Not on CRF*

4.4 Bronchial Thermoplasty?

		Start Date	Ongoing?	End Date
Yes O	→	D D / M M / Y Y	Yes O	
No O	→	*Q4.5*	No O →	D D / M M / Y Y

No, never

4.5 Thermal laminar Airflow Device?
e.g. from LASER study
Yes O → D D / M M / Y Y
No O → *Q4.6*

No

4.6 Inhaler technique: **Date technique assessed** D D / M M / Y Y
Technique: **Good** O *No changes needed* — **Sub-optimal** O *Minor changes needed* — **Poor** O *Major changes, or change of inhaler needed* — **Not done** O

Not done

4.7 Action plan:
Adequate plan pre-existing? Yes O No O — *Missing*
Action plan given? Yes O No O — *Missing*
→ **Date performed** D D / M M / Y Y

Comments ....

Page completed by ______
Date completed D D / M M / Y Y

5. Smoking History

Respnses from 21Mar2017 (Enrolment CRF)

5.1 Smoking status

- **Never smoked** O → *Q5.3*
- **Current smoker** O → **Age started** |__|__| years
- **Ex-smoker** O → **Age started** |__|__| years **Age stopped** |__|__| years

Ex-smoker
Started aged 20
Finished aged 22

5.2 Pack-year history

a) Cigarettes
- **Number smoked per day** |__|__|__| cigarettes
- **Number of years smoked** |__|__| years

b) Pipes / Cigars
- **Number smoked per day** |__|__| pipes / cigars → **Pipes** O **Cigars** O **Other** O → **Specify** |________|
- **Number of years smoked** |__|__| years

c) Cigarillos
- **Number smoked per day** |__|__| cigarillos
- **Number of years smoked** |__|__| years

d) Tobacco
- **Grams smoked per day** |__|__| . |__| gms
- **or**
- **Ounces smoked per day** |__|__| . |__| oz
- **Number of years smoked** |__|__| years

Pack-years Calculation
= Cigarettes per day x yrs smoked / 20
1 Cigar/pipe/cigarillo = 5 cigarettes
12.5g or 0.5oz tobacco = 20 cigarettes
Total pack years |________|

5.3 Passive exposure status

- **Currently** O
- **In the past** O
- **Never** O

→ **From:** **Parents?** ☐ **Spouse?** ☐ **Others?** ☐ → **Specify** |________|
Tick all that apply
Number of years |__|__| years

In the past
50 yrs

E-cigarettes

5.4 Uses e-cigarettes regularly? Yes O No O → *Next Section*

No

- **5.4.1 Used for how long?** |__|__| years |__|__| months
- **5.4.2 Nicotine dose** |__|__| mgs *(Common values are 0, 3, 6, 12, 18 and 20)*
- **5.4.3 How long does it take to use 10ml of e-liquid?** |__|__| weeks |__| days
- **5.4.4 Which flavour(s)?** |________|

Comments ....
....
....

Page completed by |________|
Date completed D D / M M / Y Y

6. Nasal Disease

Responses from 21 Mar 2017 (Enrolment CRF)

Since the last WATCH Study Visit have you...

				Responses from 21 Mar 2017 (Enrolment CRF)
**6.1 ... had rhinitis?**	**Yes** O	**No** O	**Not known** O	*Yes*
**6.2 ... had nasal polyps?**	**Yes** O	**No** O	**Not known** O	*Yes*
**6.3 ... had nasal surgery?**	**Yes** O	**No** O	**Not known** O	*Yes*

Comments ....

....

**6.4 ... had any Perennial Allergy?**	**Yes** O	**No** O	**Not known** O	*No*
**6.5 ... had any Seasonal Allergy?**	**Yes** O	**No** O	**Not known** O	*No*

Comments ....

....

6.6 ... had CT Sinuses performed? Yes O　No O　**N/K** O　*Yes; Date: 01/07/2016*
(Clinical indication only)

→ **Date performed** D D / M M / Y Y

Findings:
- ☐ **Normal**
- ☐ **Polyps**
- ☐ **Mucosal thickening**
- ☐ **Sinus opacification / abnormality**
- ☐ **Abnormal nasal turbinates**
- ☐ **Other** → **Specify** [ ]

7. Reflux History

Responses from 21 Mar 2017 (Enrolment CRF)

Since your last WATCH Study Visit have you...

7.1 ... been diagnosed with GORD? Yes O　No O　*Yes*
(Patient self-report or clinician diagnosis in letter / notes)

**7.2 ... had any symptoms of GORD?**		Responses from 21 Mar 2017
Yes, and ongoing despite on treatment	O	*Yes, and ongoing despite treatment*
Yes, but not on treatment at present	O	
No, on treatment and symptoms controlled	O	
No	O	

7.3 ... had any oesophageal testing performed or planned
(Only if clinically indicated)

	**Yes, done**	**No**	**Requested, not yet done**	**Requested, but not done for some reason e.g. DNA**	Responses from 21 Mar 2017
**a) Barium swallow?**	O → **Date** D D / M M / Y Y	O	O	O	*No/Never*
**b) OGD?**	O → **Date** D D / M M / Y Y	O	O	O	*No/Never*
**c) Oesophageal manometry PH / impedance?**	O → **Date** D D / M M / Y Y	O	O	O	*No/Never*
**d) Other?** → **Specify** [ ]	O → **Date** D D / M M / Y Y	O	O	O	*No/Never*

Page completed by [ ]

Date completed D D / M M / Y Y

8. Psychological History

Responses from 21 Mar 2017 (Enrolment CRF)

Since your last WATCH Study Visit have you...

8.1 ... had any Diagnosis* of:

			Responses from 21 Mar 2017 (Enrolment CRF)
- **Depression?**	Yes O	No O	*No*
- **Anxiety?**	Yes O	No O	*No*
- **Other psychological / psychiatric comorbidity?**	Yes O → **Specify** ____	No O	*No*

** Patient self-report or clinician diagnosis in letter / notes.*

8.2 ...been seen by mental health professionals?

	Yes	No	Requested, not yet done	Requested, but not done for some reason e.g. DNA	Responses from 21 Mar 2017 (Enrolment CRF)
**a) Psychiatrist?**	O → **Date** D D / M M / Y Y; Completed O; Ongoing O	O	O	O	*No/Never*
**b) Psychologist?**	O → **Date** D D / M M / Y Y; Completed O; Ongoing O	O	O	O	*No/Never*
**c) Specialist asthma psychologist?**	O → **Date** D D / M M / Y Y; Completed O; Ongoing O	O	O	O	*No/Never*
**d) Other?** → **Specify** ____	O → **Date** D D / M M / Y Y; Completed O; Ongoing O	O	O	O	*No/Never*

Page completed by ____

Date completed D D / M M / Y Y

9. Breathing Pattern Disorders

Since your last WATCH Study Visit have you...

		Yes	No	*Responses from 21 Mar 2017 (Enrolment CRF)*
**9.1 ... had any diagnosis of:** *(Patient self-report or clinician diagnosis in letter / notes)*	**a) Vocal cord dysfunction?**	O	O	*No*
	**b) Dysfunctional breathing?**	O	O	*Yes*

9.2 ... had any investigations for VCD?

	Yes, done	No	Requested, not yet done	Requested, but not done for some reason e.g. DNA	*Responses from 21 Mar 2017 (Enrolment CRF)*
**a) Nasendoscopy?**	O → Date D D / M M / Y Y; Completed O Ongoing O	O	O	O	*Yes; Date: 01/06/2015*
**b) Other?** → Specify [ ]	O → Date D D / M M / Y Y; Completed O Ongoing O	O	O	O	*Missing*

9.3 ... been seen by a BPD / VCD specialist?

	Yes, done	No	Requested, not yet done	Requested, but not done for some reason e.g. DNA	*Responses from 21 Mar 2017 (Enrolment CRF)*
**a) Physiotherapist?**	O → Date D D / M M / Y Y; Completed O Ongoing O	O	O	O	*No/Never*
**b) Specialist asthma physiotherapist?**	O → Date D D / M M / Y Y; Completed O Ongoing O	O	O	O	*No/Never*
**c) Speech therapist?**	O → Date D D / M M / Y Y; Completed O Ongoing O	O	O	O	*No/Never*
**d) Other?** → Specify [ ]	O → Date D D / M M / Y Y; Completed O Ongoing O	O	O	O	*No/Never*

Page completed by [ ]

Date completed D D / M M / Y Y

10. Comorbidities and Procedures

Last asked/updated 21 Mar 2017

10.1 Comorbidities

	Previously had? * Yes	No	N/K	Currently have? Yes	No	N/K
a) COPD	O	✓	O	O	O	O
b) Allergic Bronchopulmonary Aspergillosis or SAFS	O	✓	O	O	O	O
c) Non-CF Bronchiectasis	O	✓	O	O	O	O
d) Cystic Fibrosis	O	✓	O	O	O	O
e) Bronchiolitis	O	✓	O	O	O	O
f) Churg Strauss Syndrome	O	✓	O	O	O	O
g) Chronic urticaria / angioedema	O	✓	O	O	O	O
h) Sulphite sensitivity	✓	O	O	O	O	O
i) Salicylate (aspirin) sensitivity	✓	O	O	O	O	O
j) Eczema	O	✓	O	O	O	O
k) Latex allergy	O	✓	O	O	O	O
l) Sleep apnoea	O	✓	O	O	O	O

** The ticks are responses at earlier visits. Please update as necessary.*

Existing Other Comorbidities

	Previously had?	Currently have? Yes	No	N/K
RHINITIS	✓	O	O	O
GORD	✓	O	O	O
DYSFUNCTIONAL BREATHING	✓	O	O	O
OSTEOPOROSIS	✓	O	O	O
IRRITABLE BOWEL SYNDROME	✓	O	O	O

New comorbidities since last visit *(write in)*

		Previously had?	Currently have? Yes	No	N/K
1.	______	O	O	O	O
2.	______	O	O	O	O
3.	______	O	O	O	O
4.	______	O	O	O	O
5.	______	O	O	O	O
6.	______	O	O	O	O

10.2 Procedures

	Ever Received? Yes	No	N/K	If 'Yes', Date of Procedure
a) Bariatric surgery	O	O	O	D D / M M / Y Y
b) Hiatus hernia repair	O	O	O	D D / M M / Y Y
c) Hysterectomy	O	O	O	D D / M M / Y Y

Previous Other Procedures

New Procedures since last visit *(write in)*

		Ever Received?	Date of Procedure
1.	______	O	D D / M M / Y Y
2.	______	O	D D / M M / Y Y
3.	______	O	D D / M M / Y Y
4.	______	O	D D / M M / Y Y
5.	______	O	D D / M M / Y Y
6.	______	O	D D / M M / Y Y

Page completed by ______

Date completed D D / M M / Y Y

11. Anthropometry

Height: 1.58m on 21 Mar 2017 (Enrolment

Weight: 69.70kg on 21 Mar 2017 (Enrolment

11.1 Height |_|_|.|_|_| metres **How obtained?** Measured O Asked patient O

11.2 Weight |_|_|_|.|_|_| kg **How obtained?** Measured O Asked patient O

12. Imaging - HRCT

Scan last performed 21 Mar 2017 (Enrolment CRF)

12.1 HRCT performed? Yes O No O

12.2 Date performed D D / M M / Y Y

12.3 HRCT evidence of:

	Yes	No
Central bronchiectasis	O	O
Other bronchiectasis	O	O
Bronchial dilation without bronchiectasis	O	O
Bronchial wall thickening	O	O
Ground glass shadowing	O	O
Mucus plugging	O	O
Air trapping	O	O
Scan reported as normal by radiologist	O	O
Emphysema - Centrilobular	O	O
Emphysema - Panacinar	O	O
Other category / other findings	O	O

Specify in 'Comments' below

Comments ....

13. Imaging - DEXA

No record of any previous scan.

13.1 DEXA scan performed? Yes O No O

13.2 Date performed D D / M M / Y Y

13.3 Spinal bone density (L1-4) T score |______|

13.4 Femoral neck bone density (L1-4) T score: a) Left |______|

b) Right |______|

Comments ....

14. Concordance

No record of any previous results.

14.1 % Concordance preventer |_|_|_| %

14.2 Number of refills for the reliever |_|_| **per how many weeks?** |_|_| *wks*

14.3 Subjective clinical impression of concordance

Good O

Sub-optimal O

Poor O

Unable to comment O

Comments ....

Page completed by |______|

Date completed D D / M M / Y Y

At this point ask the patient to complete the Questionnaires (esp. ACQ 6/7 and EuroQoL-5D)

Questionnaires Previously Completed

CRF	ACQ 6/7		EuroQoL EQ-5D-5L		Nijmegen		Epworth Sleepiness		H A D S	
	Date	Score	Date	Score	Date	Score	Date	Score	Date	Score
Enrolment	13/03/2017	4.2	13/03/2017	313	13/03/2017	99	13/03/2017	18	13/03/2017	99

15. Dietary Assessment

No previous record.

15.1 Body composition assessed? **Yes** O **No** O → *Q15.2*

Test	Result	
FFM		kg
FM		kg
FFMi		kg/m2
SMM		kg
PhA		degrees

15.2 Seen by asthma specialist dietician?

Tick ONE only

- **Not required** O
- **Ongoing** O
- **To be seen** O
- **Discharged, treated** O
- **Discharged, DNA** O

16. Research Samples

**16.1 Date research bloods taken**	D D / M M / Y Y	*Collected 21Mar 2017*
**16.2 Date urine taken**	D D / M M / Y Y	*Collected 21 Mar 2017*
**16.3 Date sputum taken**	D D / M M / Y Y	*Not yet collected*

16b. Research Samples for Patients Enrolled in "WATCH NIH" Study

**16b.1 Date PBMC #1**	D D / M M / Y Y	*Not yet collected*
**16b.2 Date PBMC #2**	D D / M M / Y Y	*Not yet collected*
**16b.3 Date PBMC #3**	D D / M M / Y Y	*Not yet collected*
**16b.4 Date Sputum**	D D / M M / Y Y	*Not yet collected*
**16b.5 Date Bronch**	D D / M M / Y Y	*Not yet collected*

Page completed by ______

Date completed D D / M M / Y Y

17. FeNO (Exhaled Nitric Oxide)

Test last performed 21 Mar 2017 (Enrolment CRF)

17.1 Was FeNO(50) done?

Yes O No O → *17.2*

→ **17.1.1 Date performed** D D / M M / Y Y

17.1.2 Where done? **UHS PFT** O **UHS Research Lab.** O **IOW OPD Clinic** O **UHS Resp. Centre** O **Portsmouth OPD Clinic** O

17.1.3 Did the patient refrain from eating and drinking for at least 2 hours prior to the FeNO measurement? **Yes** O **No** O **Unknown** O

→ *Consider performing test at next visit*

17.1.4 Bedfont (50ml/sec) **1.** ____ **2.** ____ **3.** ____

17.2 Was Multiple Flow NO done? *(University Hospital Southampton only)*

No record of any previous test.

Yes O No O → *Next Section*

→ **17.2.1 Date performed** D D / M M / Y Y

17.2.2 Test results *Attach the results printout to this CRF.*
Please ensure the Subject No. is written clearly on the printout.

Comments ....

18. Spirometry

No record of any previous test.

18.1 Was Spirometry done? Yes O → *18.2* No O → *Next Section*

Use historical REVERSIBILITY data if available within last 12 months

18.2 Date of test D D / M M / Y Y

18.3 Where done? **UHS PFT** O **UHS Research Lab.** O **IOW OPD Clinic** O **UHS Resp. Centre** O **Portsmouth OPD Clinic** O

18.4 Was acceptable spirometry obtained? **Yes** O **No** O
(Only record parameters of good quality)

18.5 Was a washout period observed? **Yes** O **No** O → *Record results as POST-BD*
(SABA 4 hours and LABA 12 hours)

18.6 Last use of short-acting β-agonist **Date** D D / M M / Y Y **Time** H H : M M
(Salbutamol, Ventolin, Terbutaline, Bricanyl)
or NA ☐ *(e.g. if using maintenance inhaler as reliever)*

18.7 Last use of long-acting β-agonist **Date** D D / M M / Y Y **Time** H H : M M
(Formoterol, Fumarate, Salmeterol, Bambuterol)

18.8 Test results *Attach the results printout to this CRF.*
Please ensure the Subject No. is written clearly on the printout.

Comments ....

Page completed by ____

Date completed D D / M M / Y Y

19. Lung Volumes

No record of any previous test.

19.1 Date of test D D / M M / Y Y

19.2 Where done? UHS PFT O UHS Research Lab. O IOW OPD Clinic O Portsmouth OPD Clinic O

19.3 Method used Plethysmography O Nitrogen washout O Other O → Specify ______

19.4 Test results *Attach the results printout to this CRF. Please ensure the Subject No. is written clearly on the printout.*

Comments ....

20. Single Breath Diffusion / Transfer Facto

No record of any previous test.

20.1 Was test done?

Yes O No O → *Next Section*

→ **20.2 Date of test** D D / M M / Y Y

20.3 Where done? UHS PFT O UHS Research Lab. O IOW OPD Clinic O Portsmouth OPD Clinic O

20.4 Test results *Attach the results printout to this CRF. Please ensure the Subject No. is written clearly on the printout.*

Comments ....

21. Impulse Oscillometry (IOS)

No record of any previous test.

21.1 Was test done? Yes O No O → *Next Section*

If Yes:

21.2 Date of tests D D / M M / Y Y

21.3 Where done? UHS PFT O UHS Research Lab. O IOW OPD Clinic O Portsmouth OPD Clinic O

21.4 Was a washout period observed? *(SABA 4 hours and LABA 12 hours)* **Yes** O **No** O → *Record results as POST-BD*

21.5 Last use of short-acting β-agonist *(Salbutamol, Ventolin, Terbutaline, Bricanyl)* **Date** D D / M M / Y Y **Time** H H : M M

or NA ☐ *(e.g. if using maintenance inhaler as reliever)*

21.6 Last use of long-acting β-agonist *(Formoterol, Fumarate, Salmeterol, Bambuterol)* **Date** D D / M M / Y Y **Time** H H : M M

21.7 Test results *Attach the results printout to this CRF. Please ensure the Subject No. is written clearly on the printout.*

Comments ....

Page completed by ______

Date completed D D / M M / Y Y

22. Multiple Breath Nitrogen Washout (MBNW) *(University Hospital Southampton only)*

No record of any previous test.

22.1 Was test done? Yes O No O → *Next Section*

22.2 Date of test D D / M M / Y Y

22.3 Time of test H H : M M

22.4 Test Results *Attach the results printout to this CRF.*
Please ensure the Subject No. is written clearly on the printout.

Comments ....

23. Allergy Testing

Last tested 21Mar 2017 (Enrolment CRF)

23.1 Skin prick test form attached?
Yes O No O

23.2 Date performed D D / M M / Y Y

23.3 Number of days H1 or H2 anti-histamines omitted? n/a ☐ or ___ days
(If greater than 3 days just enter '3')

23.4 Number of days Tricyclic Antidepressants (TCAs) omitted? n/a ☐ or ___ days
(If greater than 7 days just enter '7')

Comments ....

24. Allergy RAST Testing

24.1 Previous Tests

24.2 New tests carried out since last WATCH Study visit

	Allergen	Test Date	Result	Grade	Notes
1.		D D / M M / Y Y			
2.		D D / M M / Y Y			
3.		D D / M M / Y Y			
4.		D D / M M / Y Y			
5.		D D / M M / Y Y			

Page completed by ______

Date completed D D / M M / Y Y

25. Sputum

No record of any previous sputums.

25.1 Sputum eosinophils |__|__|__| %

25.2 Sputum neutrophils |__|__|__| %

26. Bronchoscopy

No record of any previous bronchoscopy.

26.1 Bronchoscopy performed? *(Only if clinically indicated)*

Yes O No O

→ **26.2 Date performed** D D / M M / Y Y

26.3 Histological findings? Yes O No O

→ *(Attach printout report)*

26.4 Brushings and Biopsies

	Number	Time	Site of Samples
a) Biopsies		HH:MM	
b) Brushings		HH:MM	

26.5 Bronchial lavage

In |__|__| ml **Out** |__|__| ml

Comments ....

27. Other Research Study Participations

Set in Code

- a) WATCH BI ☐
- b) WATCH NIH ☑
- c) WATCH ROGSA ☐
- d) Panos ☐
- e) WSAC ☐
- f) UBIOPRED ☐
- g) SoMOSA ☐
- h) RASP UK ☐
- i) RASP Bronch ☐
- j) MEPO ☐
- k) MIDAS ☐

Others (specify)

- i ________ ☐
- ii ________ ☐
- iii ________ ☐
- iv ________ ☐

Page completed by ________

Date completed D D / M M / Y Y
