# Supplementary material for: Protocol for the Wessex AsThma CoHort of difficult asthma (WATCH): a pragmatic real-life longitudinal study of difficult asthma in the clinic
Source: BMC Pulm Med. 2019 May 24;19:99. doi: 10.1186/s12890-019-0862-2 (PMC6534885; doi:10.1186/s12890-019-0862-2)
Supplement: Supplementary file 3 — Appendix A Clinical Investigations. Appendix B Lung Function Test. Appendix C Biobank Samples. (DOCX 25 kb) [file 12890_2019_862_MOESM3_ESM.docx]

# Additional file 3

# Appendix A: Clinical Investigations

### Anthropometry

Height to within 0.01 metres and body weight to within 0.01 kilograms are measured at each study visit. Body Mass Index (BMI) (kg/m^2^) is calculated from these values. Bioelectrical Impedance Analysis (BIA) is performed on all participants to assess body composition. This method is based on the conduction of electrical current in the body and differences in electrical conductivity between the fat and water components of the body. The electrical resistance and reactance together with body weight and height can reliably estimate body composition. Participants stand on the Seca mBCA (Medical Body Composition Analyser): each foot on a pair of electrodes and each hand on electrodes within the handrails. The mBCA measures impedance at a number of frequencies ranging from 1 KHz to 1 MHz. BIA derived measures are generated according to manufacturer equations. These measures include Fat Free Mass (FFM) (kg), Fat Mass (FM) (kg), Free Fat Mass Index (FFMi) (kg/m^2^), Skeletal Muscle Mass (SMM) (kg) and Phase Angle (PhA) (degrees), where the latter is a good indicator of cell membrane function as well as a marker of nutritional status.

### Historical Blood Tests

Clinically requested blood tests (see table 4) are processed by the UHSFT Pathology Laboratory, which is fully UKAS (United Kingdom Accreditation Service) accredited and compliant to ISO142819 standards. Results are stored on the hospital’s local pathology results server from which data is extracted (see data management). Rarely, if the patient has had blood test results at another site (e.g. local secondary care hospital), these are sought and imported to the study database.

### Skin Prick Testing

Skin prick testing is usually performed at first clinical assessment to a standard panel of common aeroallergens by the Asthma Specialist Nurses using standard commercially available allergen extract solutions. This includes positive (histamine) and negative (saline) controls plus Aspergillus Fumigatus; Altenaria Tenius; Cladosporium; Grass Mix Pollen; Birch Pollen; Weed Mix (Mugworth, Nettle, Pellion, Dandelion, English Plantain); Flower Mix (Aster, Chrysanthemum, Dahlia, Golden Rod, Marguerite); Rape Pollen; Dermatophagoides pteronyssinus; Dermatophagoides farinae; Feathers; Cat Fur; Dog Fur; Horse and Rabbit.

Antihistamines are omitted 3 days prior to the test and tricyclic antidepressants 7 days prior to the test. A positive skin prick test is defined as a mean wheal diameter of ≥3mm than the negative control.

### Radiology Results

Clinically requested radiological investigations are usually performed by the UHSFT Radiology Department, which operates according to Royal College of Radiology guidelines and standards. Results are stored on the hospital’s local radiology results server (e.g. PACS) from which data is extracted (see data management). If the patient has had imaging results at another site (e.g. local secondary care hospital), then these are sought and imported to the study database.

### Oesophageal Investigation Results

Clinically requested oesophageal investigations are performed by the UHSFT Endoscopy Department, which operates according to the Joint Advisory Group on Gastrointestinal Endoscopy guidelines and standards. Results are stored on the hospital’s local results server from which data is extracted (see data management). If the patient has had oesophageal investigations at another site (e.g. local secondary care hospital), then these results are sought and imported to the study database.

### Nasoendoscopy Results

Clinically requested flexible nasoendoscopies are performed by the ENT team at UHSFT using a standard three pass technique. Results are recorded in the electronic correspondence server from which data is extracted (see data management). If the patient has had nasoendoscopy results at another site (e.g. alternative tertiary care service), these are sought and imported to the study database.

# Appendix B: Lung Function Test

Subjects were asked to refrain from the following, if possible, before lung function testing:

- No smoking for 24 hours
- No alcohol consumption for 4 hours
- No vigorous exercise for 30 minutes
- No tight fitting clothing that could restrict full chest and/or abdominal expansion
- No food for 2 hours
- If clinically acceptable, no supplemental oxygen for 10 minutes

### Spirometry with Reversibility

For spirometry, if patients are able to withhold their regular inhaler therapy then reversibility testing was performed using salbutamol (2.5mg nebulised or 400µg inhaled via a spacer device), to give a “pre-bronchodilator“ and “post-bronchodilator“ measurement.

Restrictions before Reversibility Testing

• Withhold short-acting inhalers such as the ß_2_-agonist salbutamol or the anticholinergic drug ipratropium bromide for at least 4 hours

• Withhold long-acting ß_2_-agonist bronchodilators such as salmeterol for at least 12 hours

- Withhold oral therapy with aminophylline or slow-release ß-agonists for 12 hours

Spirometry was performed using either Carefusion® (Chatham, UK) or nSpire Health Ltd (Hertford, UK) equipment according to ERS/ATS guidelines [1]. If subjects were unable to withhold regular inhaler therapy then spirometry results were recorded as “post-bronchodilator”. Z Scores and percentage predictive values were calculated using the Global Lung Function Initiative (GLI) look up tables.

### Exhaled Nitric Oxide

Fractional Exhaled Nitric Oxide (FeNO) was measured using the NIOX VERO® (Oxford, UK) or Bedfont NObreath® (Aylesford, UK), at a flow rate of 50ml/s according to ERS/ATS guidelines [2]. Exhaled nitric oxide was measured first prior to any other lung function test due to the influence of breathing manoeuvres on FeNO readings. A minimum of two technically acceptable tests was recorded, with the two highest values within 10% of each other and the mean value reported.

### Gas Transfer

Gas transfer was measured using the single breath method and the nSpire HDpft 4000 (Hertford, UK) according to ERS/ATS guidelines [3] with results recorded in SI units (mmol.min^-1^.kPa^-1^). At least two technically acceptable and repeatable TLCO measures were recorded and the mean reported. There was a 4 minute wait between tests and no more than 5 tests were performed. Z Scores and percentage predictive values were calculated using the equations outlined in the ERS 1993 recommendations.

### Impulse Oscillometry

Impulse oscillometry was measured using the Jaeger Impulse Oscillation System, Carefusion® (Chatham, UK) and performed according to ERS/ATS guidelines [4]. Subjects supported their cheeks with their hands and breathed normally until at least 90 seconds of data was recorded. This was either done in three trials of 30 seconds or one trial of 90 seconds. The breathing pattern was observed for consistency and to check it was free of artefacts. If three trials were recorded then the average of the trials was used for each index. Percentage predictive values were calculated using the Vogel 1994 equations [5].

### Static Lung Volumes

Lung volume measurements were performed using either body plethysmography or nitrogen washout on the nSpire HDpft 4000. For plethysmography, at least three technically acceptable and repeatable functional residual capacity (FRC) measurements were obtained and the mean values of the indices reported. For static lung volumes using single breath nitrogen washout, at least one technically acceptable test was performed and this value used. If more than one test was done, the FRC values were within 10% of each other and the mean reported. Z Scores and percentage predictive values were calculated using the equations outlined in the ECSC 1993 recommendations [6].

### Multiple Breath Nitrogen Washout

Multiple breath nitrogen washout was performed by research staff at study visits using local SOPs in accordance with ERS/ATS guidelines [7]. It was measured using the Ecomedics Exhalyzer D (*Dürnten, Switzerland*). At least two technically acceptable (LCI_2..5_) tests were achieved and the mean reported. For LCI_2..5_, this involved the subject breathing 100% oxygen tidally until at least three breaths under the target nitrogen value was achieved. If only S_acin_ and S_cond_ were measured, tidal breathing until at least 6 turnovers was required. A waiting period >1.5 times the time taken to washout was observed between tests.

# Appendix C: Biobank Samples

### Blood Samples

Research blood samples are taken at enrolment where possible for our biobank: collected in purple capped EDTA (ethylenediaminetetraacetic acid) tubes, gold capped SST (serum separating tubes – containing clot activator *and* serum separating gel) and red capped tubes (no additives or separating gel). Blood is processed as soon as possible within 30-60 minutes of collection.

*Whole Blood:* Whole blood collected in an EDTA tube is labelled and stored at below -70 ˚C.

*Plasma:* Whole blood from an EDTA bottle is centrifuged for 15 minutes at 1750 xG at 4˚C. Plasma is then pipetted into polypropylene microtubes, labelled and stored at below -70 ˚C.

*Serum:* Whole blood from a red capped tube is left to clot for 30-60 minutes. It is then centrifuged for 15 minutes at 1750 xG at 4˚C. Serum is then pipetted into polypropylene microtubes, labelled and stored at below -70 ˚C. Whole blood from a gold capped serum separator tube containing the clotting agent silica is left to clot for 30-60 minutes. It is then centrifuged for 15 minutes at 1750 xG at 4˚C. Serum is then pipetted into polypropylene microtubes, labelled and stored at below -70 ˚C.

### Urine Samples

Urine is processed as soon as possible and within 2 hours of production. It is centrifuged for 10 minutes at 790 xG at 4˚C. Aliquots are stored in 1 ml, 5ml and bulk volumes.

### Sputum Induction

Clinically indicated sputum Induction is performed by the research team in the Environmental Chambers of the Clinical Research Facility at UHSFT, using a DeVilbiss 2000 Ultrasonic Nebuliser (Somerset, USA) and protocols based on those described by the ERJ Working Group (10.1183/09031936.02.00000902).

Spirometry with reversibility is recorded prior to the procedure (See Lung Function Testing). Three 5 minute rounds of saline nebulisation, starting at 0.9% and escalating to 3% then 4.5% are performed with FEV1 measurements after each round. Patients undergo a modified protocol (2 minutes nebulisation) if their FEV1 <1.5L. Should the participant need to cough and expectorate during the nebulisation then they can do so and collect any dislodged sputum into a petri dish.

Sputum is processed as soon as possible and within 2 hours of expectoration. Sputum plugs are visually identified, selected and condensed into one mass to remove contaminating saliva and homogenised. The extent of processing will depend upon the amount of sputum obtained however the concurrent method described by Bafadhel will be used where possible [8]:

1. Dithiothreitol (DTT) only (*Sigma Aldrich, Dorset, UK*)
2. DTT and Phosphate Buffered Saline (PBS) (*Fisher Scientific, Leicester, UK*)
3. DTT, PBS and Bacteriology Sample

*Bacteriology:* If there is sufficient sample, 0.05 – 0.10g of the sputum plug is stored raw

*PBS Processing:* If there is sufficient sample, PBS processing is performed. Sputum is incubated on ice with 8 volumes of PBS and passed through a pasteur pipette, vortexed and then agitated on ice at 4˚C on a bench roller for 30 minutes. The sample is then centrifuged and 6 volumes of supernatant is removed and centrifuged again at a higher speed to produce PBS supernatant aliquots for storage at below -70 ˚C.

*DTT Processing:* Two volumes of 0.2% DTT is added to the remaining sample, giving a final concentration of 0.1% DTT. The samples is again passed through a pasteur pipette, vortexed and subsequently agitated on ice at 4˚C on a bench roller for 30 minutes. The sample is then centrifuged and DTT supernatant aliquots are stored at below -70 ˚C.

The cell pellet is re-suspended in PBS and counted using trypan blue exclusion method to ascertain the number of live and dead cells. Cytospins are generated using a Shandon Cytospin 2 and remaining cells are stored in RNAlater stabilisation and storage solution (*Ambion, USA*). Two cytospins are stained using rapid Romanowski stain and 400-800 respiratory cells (e.g. neutrophils, eosinophils, macrophages, lymphocytes and columnar epithelial cells) plus squamous cells are counted for differential cell count and to ascertain the volume of squamous cell contamination.

### Bronchoscopy

Clinically indicated bronchoscopy is performed by the research team in the Bronchoscopy Suite of the Clinical Research Facility at UHSFT according to BTS Standards (ISSN 2040-2023).

*Bronchoalveolar lavage (BAL):* The bronchoscope is wedged into the right upper lobe and 20ml of warmed 0.9% saline is added to the lungs. The 0.9% saline is rested for 10 seconds and then aspirated into a storage vessel. This is repeated until 100-120ml of 0.9% saline has been used for the wash. BAL is stored on ice and processed as soon as possible within 30 minutes of collection. BAL volume returned is recorded and a small sub aliquot is taken for microbiological analysis. The remaining sample is passed through a 100μM cell strainer and then centrifuged. The cells are re-suspended and counted using the trypan blue exclusion method to ascertain the number of cells recovered and their viability. Any visible appearance of red blood cell contamination during BAL is noted. Cytospins are generated using a Shandon Cytospin 2 and remaining cells are stored in RNAlater solution. Two cytospins are stained using rapid Romanowski stain and 400-800 respiratory cells plus squamous cells are counted for differential cell count and to ascertain the volume of squamous cell contamination.

*Bronchial biopsies:* Biopsies are stored in 10% formalin overnight at 2 to 8 ˚C. These are then embedded in paraffin blocks and stored at room temperature. The tissue containing blocks are sliced using a microtome and the slices are placed onto a microscope slide with subsequent staining using immunohistochemistry.

## References

1. Miller MR, Hankinson J, Brusasco V, Burgos F, Casaburi R, Coates A, et al. Standardisation of spirometry. Eur Respir J. 2005;26:319-38. doi: 10.1183/09031936.05.00034805.

2. American Thoracic S, European Respiratory S. ATS/ERS recommendations for standardized procedures for the online and offline measurement of exhaled lower respiratory nitric oxide and nasal nitric oxide, 2005. Am J Respir Crit Care Med. 2005;171:912-30. doi: 10.1164/rccm.200406-710ST.

3. Macintyre N, Crapo RO, Viegi G, Johnson DC, van der Grinten CP, Brusasco V, et al. Standardisation of the single-breath determination of carbon monoxide uptake in the lung. Eur Respir J. 2005;26:720-35. doi: 10.1183/09031936.05.00034905.

4. Oostveen E, MacLeod D, Lorino H, Farre R, Hantos Z, Desager K, et al. The forced oscillation technique in clinical practice: methodology, recommendations and future developments. Eur Respir J. 2003;22:1026-41. https://www.ncbi.nlm.nih.gov/pubmed/14680096.

5. Vogel J SU. Impulse Oscillometry. Analysis of lung mechanics in general practice and clinic, epidemiological and experimental research: Frankfurt am Main, Pmi-Verlagsgruppe; 1994.

6. Wanger J, Clausen JL, Coates A, Pedersen OF, Brusasco V, Burgos F, et al. Standardisation of the measurement of lung volumes. Eur Respir J. 2005;26:511-22. doi: 10.1183/09031936.05.00035005.

7. Robinson PD, Latzin P, Verbanck S, Hall GL, Horsley A, Gappa M, et al. Consensus statement for inert gas washout measurement using multiple- and single- breath tests. Eur Respir J. 2013;41:507-22. doi: 10.1183/09031936.00069712.

8. Bafadhel M, McCormick M, Saha S, McKennaS, Shelley M, Hargadon B, Mistry V, Reid C, Parker D, Dodson P, Jenkins M, Lloyd A, Rugman P, Newbold P, Brightling C. Profiling of Sputum Inflammatory Mediators in Asthma and Chronic Obstructive Pulmonary Disease.

Respiration, 2012;83(1)36-44 doi 10.1159/000330667
